# Non-cell-autonomous manner of AAV administration to attenuate cardiomyocyte hypertrophy by targeting paracrine signaling on ECM to reduce viral dosage

**DOI:** 10.1038/s41392-021-00715-z

**Published:** 2022-01-03

**Authors:** Lei Liu, Peng Yue, Yue Zhang, Yimin Hua, Wenwei Bi, Hualin Yan, Hongyu Liao, Jiawen Li, Kaiyu Zhou, Yifei Li

**Affiliations:** 1 Key Laboratory of Birth Defects and Related Diseases of Women and Children of MOE, Department of Pediatrics, West China Second University Hospital, Sichuan University, Chengdu, Sichuan China; 2 Institute of Neuroscience and Medicine 4, INM-4, Forschungszentrum Jülich, Jülich, Germany; 3 Department of Medical Ultrasound, West China Hospital, Sichuan University, Chengdu, Sichuan China

**Keywords:** Non-coding RNAs, Cardiology

**Dear Editor**,

Adeno-associated virus (AAV) is one of the most powerful vectors for exogenous gene delivery.^1^ However, the risks associated with the high dosages of AAV administration that are required to achieve meaningful effects limit the applicability of this method. Generally, AAV only presents cell autonomous manner in infected cells. Besides, mosaic genetic editing or gene therapy would trigger global biological function restore by above 30–40% infective ratio in heart diseases.^2^ However, if non-cell-autonomous effect could be achieved for AAV gene therapy, the viral dosage for AAV administration would reduced significantly, avoiding high dosage risks. Extracellular matrix (ECM) remodeling is a response to maladaptive stimuli, especially for exceeding mechanical stress.^3^ Increased stiffness of myocardial tissue ECM is associated with enhanced mechanotransduction and activation of integrin/Hippo/Yap signaling.^4^ Several cytokines and molecules are involved in ECM remodeling by paracrine manner, including CTGF. Accordingly, we speculated it might be potentially a non-cell-autonomous target to attenuate cardiac hypertrophy by inhibiting *Ctgf* based on its paracrine character to reduce the dosage of AAV. Besides, micro (mi)RNAs regulate many biological processes and *Ctgf* transcription,^3,5^ which is also a perfect nucleotide sequence for AAV delivery. To test this hypothesis, we developed a strategy for AAV-mediated delivery of miRNA targeting *Ctgf* to reverse the ECM remodeling to attenuate CM hypertrophy and attempt to calculate the minimum dosage of AAV required to achieve a biological therapeutic effect.

We established a mouse model of pressure overload-induced hypertrophy by abdominal aorta contraction (AAC). Significant increases in the cell area of cardiomyocytes (CMs) (Fig. 1a, b) and thickness of the left ventricular posterior wall (LVPW) were observed by echocardiography (Fig. 1c). RNA sequencing (RNA-seq) revealed overexpression of the hypertrophy-associated genes *Nppa* and *Myh7* (Supplementary Fig. 1a), which was positively correlated with increased levels of the ECM remodeling-related genes *Tgfb1*, *Tgbr2*, and *Ctgf* (Supplementary Fig. 1b, c). Enriched GO and Reactome terms were related to ECM and integrin-mediated mechanotransduction pathway components and mitochondrial dysfunction (Supplementary Fig. 2a, b). A heatmap of differentially expressed genes (Supplementary Fig. 2c) showed that Hippo signaling was the major activated pathway downstream of integrin mechanotransduction (Supplementary Fig. 2d).

**Fig. 1:**
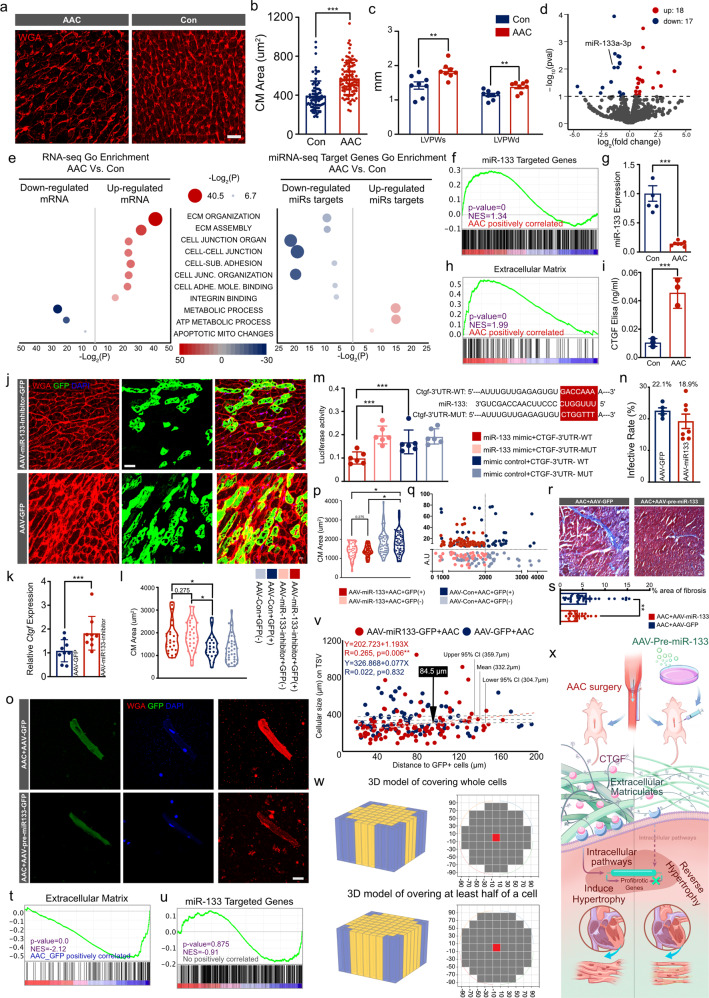
Exceeding mechanical stress induced CM hypertrophy via miR-133-CTGF-ECM-integrin-Hippo-YAP mechanotransduction signaling. Targeting ECM remodeling through miRs based therapy attenuated hypertrophic phenotype, and AAV strategy delivering miR-133 could prevent pressure overload-induced hypertrophy both in infected and surrounding non-infected CMs, which indicated a modified AAV administration approach with a reduced dosage down to a theoretic calculating value as 1.6% of infected rate to achieve therapeutic propose. **a**, **b** The WGA staining results of heart tissues (*N* = 5 biologically independent samples), and the cell size was measured in 10 fields/slice in both groups (*n* > 20 CMs for each individual animal). **c** The echocardiographic results of AAC mice demonstrated the thickness of LVPW both in systolic and diastolic period. **d** Volcano plot showing the log fold changes and log *p*-value of each gene. Up-regulated genes were marked red and down-regulated genes were marked as blue. And miR-133a-3p ranked one of the most differentiated genes. **e** Scatter plot displaying the differentiated Go term enrichments in AAC hearts of RNA-seq and differentiated miRs targeting genes of miR-seq. **f** GSEA with the genes as miR-133 targets revealed up-regulated enriched in AAV hearts. **h** Another GSEA indicted the gene ontology gene-sets of ECM-related pathway up-regulated enriched in AAC hearts. **g** qPCR demonstrated the inhibition of miR-133 in AAC hearts. **i** CTGF was significantly elevated by Elisa Assay. **j**–**l** Hypertrophy of CMs had been recorded both in infected and non-infected cells after AAV-miR-133-inhibitor-GFP administration (*N* = 5 biologically independent samples, *n* > 20 CMs for each individual animal). **k** *Cgft* expression up-regualted after miR-133 inhibition. **m** The putative binding sites located in the 3′ UTR of *Ctgf* and the experimental design of luciferase reporter. H293T cells were co-transfected with Ctgf-WT or Ctgf-MUT and miR-133 or miR-NC. The luciferase activity was analyzed. **n** Isolated CMs were measured cellular size after AAV-pre-miR-133-GFP administration. **o** Infected rated were calculated in heart cryo-sections (*N* = 4 biologically independent samples). **p**, **q** Violin and scatter plots showing the cellular area distribution with differentiated GFP density. Results revealed the attenuation of hypertrophy both in GFP+ and GFP− CMs (*N* = 5 biologically independent samples, *n* > 20 CMs for each individual animal). **r**, **s** Masson staining showing the reduction of fibrotic area after AAV-pre-miR-133 administration (*N* = 3 biologically independent samples). **t** GSEA demonstrated the gene ontology gene-sets of ECM-related pathway dysregulation in AAV delivering miR-133 hearts based on RNA-seq. **u** GSEA showed no positive correlation of miR-133 targeted genes in Yap1-overexpression hearts. **v** Linear regression to identify the distance of one infected to affect surrounding CMs. And a distance of 84.5 μm was calculated as the efficient distance to reverse hypertrophy among surrounding CMs. **w** Analysis models had been built to demonstrate the affecting surrounding area with a radius of 84.5 μm, following two kinds of principles as covering whole cells or at least half of a cell, which indicated a minimal infected rate of 1.67% or 0.64% to achieve efficient heart AAV based therapy in theory, respectively. **x** Graphic abstract was presented. Scale Bar, 50 μm; all the data were shown as mean ± SD, *P < 0.05; **P < 0.01; ***P < 0.001. AAC abdominal aorta contraction, AAV adeno-associated virus, CI confidence interval, CM cardiomyocyte, ECM extracellular matrix, LVPWs left ventricular partial wall, systolic, LVPWd left ventricular partial wall, diastolic, NES normalized enrichment score, WT wild type, Yap1-OE Yap1 overexpression

We identified 16 up-regulated and 17 down-regulated miRNAs (adjusted *p* < 0.05) by miRNA sequencing (Fig. 1d). A scatterplot analysis confirmed ECM remodeling and integrin-mediated mechanotransduction signaling up-regulated enriched in AAC hearts (Fig. 1e). Moreover, the targeting genes of down-regulated miRs were inconsistent with the results of up-regulated genes and up-regulated miRs were predicted targeting mitochondrial genes which is down-regulated in RNA-seq results. MiR-133a-3p has been retrieved in miR-seq (Supplementary Fig. 3a). There were 40 shared genes between miR-133 targeting genes and differentiated ones recorded by RNA-seq (Supplementary Fig. 3b). And ECM organization ranked the most enriched genes in the co-shared genes (Supplementary Fig. 3c). Gene Set Enrichment Analysis (GSEA) revealed that miR-133 target genes were associated with AAC (Fig. 1f), and the downregulation of miR-133 in AAC hearts was confirmed by quantitative PCR (Fig. 1g). The GSEA also showed that AAC was significantly associated with ECM organization (Fig. 1h) and increased connective tissue growth factor (CTGF) expression (Fig. 1i), implying that miR-133 contributes to ECM remodeling.

To determine whether aberrant expression of miR-133 contributes to CM hypertrophy, we used a cTnT promoter AAV vector to inhibit pre-miR-133 (Supplementary Fig. 4a). AAV-miR-133-inhibitor or AAV-green fluorescent protein (GFP) virus at 5 × 10^10^ vg/g was subcutaneously injected into mice on postnatal day 0. AAV injection did not affect postnatal growth (Supplementary Fig. 4b), but miR-133 level was decreased by ~70% in such mice (Supplementary Fig. 4c). The infection rates of miR-133–inhibited and control hearts were 38.8% and 36.3%, respectively (Supplementary Fig. 4d, e). Hypertrophic changes in the heart were detected by echocardiography in the former group (Supplementary Fig. 4f, g), which was accompanied by upregulation of hypertrophy-associated genes (Supplementary Fig. 4h) including *Ctgf* (Fig. 1k) as well as increased fibrosis (Supplementary Fig. 4i, j). We also confirmed that GFP+ CMs were much larger in miR-133–inhibited mice. Interestingly, GFP− CMs in mice treated with AAV-miR-133-inhibitor also showed signs of hypertrophy, presenting a non-cell-autonomous manner (Fig. 1j, l and Supplementary Fig. 4k). Taken together, above data suggest that inhibiting miR-133 induces cardiac hypertrophy by activating ECM remodeling.

Then we constructed wild-type and mutant *Ctgf* 3′ untranslated region (UTR) plasmids using the PEZx-FR02 vector (Fig. 1m) to confirmed *Ctgf* is the target of miR-133 according to previous publication and predicted database (miRDB and TargetScan, Supplementary Data 1 and 2). In the luciferase reporter assay, cotransfection of miR-133 mimic suppressed luciferase activity in the *Ctgf*-3′-UTR-wt group but not in the *Ctgf*-3′-UTR-mutant group (Fig. 1m). Thus, miR-133 can suppress the expression of *Ctgf* by directly targeting its 3′-UTR.

We further evaluated whether miR-133 overexpression could attenuate hypertrophy. We constructed and injected AAV-cTnT-pre-miR-133 (Supplementary Fig. 5a) along with the control virus (AAV-GFP) at a reduced dose of 2 × 10^10^ vg/g into mice at P28 to achieve a lower infection rate (Fig. 1n). AAV injection did not cause growth retardation (Supplementary Fig. 5b) but miR-133 level was about fivefold higher relative to the control (Supplementary Fig. 5c), which was accompanied by decreased expression of *Ctgf* (Supplementary Fig. 5d) and reduced LVPW thickness by echocardiography (Supplementary Fig. 5e). Additionally, the cell area was markedly smaller in isolated CMs (Fig. 1o-p) and heart tissue sections (longitudinal sections in Fig. S5F, G and transverse sections in Supplementary Fig. 6a, b) of pre-miR-133–treated mice. Moreover, the cell area of both GFP+ and GFP− CMs (Fig. 1q) as well as the fibrotic area (Fig. 1r, s) were reduced in miR-133–treated mice, suggesting a non-cell-autonomous manner of AAV therapy of miR-133 targeting CTGF paracrine effects. We performed RNA-seq to underline changes in the gene expression profile subjected to AAV-pre-miR-133 administration in AAC mice (Supplementary Fig. 7a). GSEA revealed that the expression levels of miR-133 target, ECM-related, and embryonic heart genes were decreased whereas those of adult heart genes were increased (Fig. 1t and Supplementary Fig. 7b–d). And the Hippo signaling was inhibited by pre-miR-133 administration (Supplementary Fig. 8a). Scatterplots indicated reduced enrichment of both ECM- and integrin-related GO terms and restoration of mitochondrial function (Supplementary Fig. 8b).

Given that CM hypertrophy could be attenuated by targeting ECM remodeling gene *Ctgf* using its paracrine character to achieve AAV non-cell-autonomous manner. Then we investigated whether attenuate intracellular signaling in absence of paracrine character would only demonstrate cell-autonomous manner in infected CMs. Yap1^flox/flox^ mice were subjected to AAC. Cell area was reduced only in GFP+ CMs, indicating a cell-autonomous manner (Supplementary Fig. 9a, b). Conversely, cell size was increased in primary cultures of *Yap1*-overexpressing CMs that were derived from H11em1Cin^CAG-lsl-Yap1^ mice and infected with Ad-Cre (Supplementary Fig. 9c, d). Scatterplots from GSEA based on RNA-seq data indicated that mitochondrial dysfunction was associated with Yap1 overexpression in CMs, which was similar to the AAC group; meanwhile, no significant enrichment of ECM and integrin activation was found among GO terms (Supplementary Fig. 10a), and there were also no correlations with miR-133 target genes (Fig. 1u). Besides, YAP was activated in miR-133–inhibited CMs (Supplementary Fig. 11) and decreased in those overexpressing miR-133 (Supplementary Fig. 12) underline a non-cell-autonomous manner. It is worth noting that the proportion of GFP+ cells in liver tissue was <1% following injection of a normal AAV dose (Supplementary Fig. 13).

In order to determine the paracrine affected area around infected CMs, we performed linear regression analysis between the distance to an infected CM and its cell area, which yielded the equation Y = 202.723 + 1.193X (*R* = 0.265, *p* = 0.006); in the AAC group, the equation was *Y* = 326.868 + 0.077X (*R* = 0.022, *p* = 0.832). The average area of hypertrophic CMs was 332.2 μm^2^, with a lower 95% confidence interval of 304.7 μm^2^. A value of 84.5 μm was obtained at the intersection of this value and the regression line for miR-133–attenuated hypertrophy, indicating that the effective distance of AAV-pre-miR-133 non-cell-autonomous manner was 84.5 μm (Fig. 1v). Finally, we constructed a model to determine the number of cells that were affected by a single infected CM using a Python script. We modeled a CM as a cuboid (120 × 20 × 20 μm) and calculated whether the whole CM or at least the center of the CM should be within the radius of 84.5 μm to the margins of the infected CM (Fig. 1w). The minimum infection rate for covering the whole cell was 1–60 (1.67%) and that for covering at least half of a cell was 1–156 (0.64%). Thus, in theory, the therapeutic effect can be achieved at an infection rate as low as 1.67%.

In conclusion, we identified the miR-133/CTGF/ECM/integrin/Hippo/YAP axis as an important regulator of CM hypertrophy under excessive pressure load. We also demonstrated the non-cell-autonomous manner of AAV administration by targeting paracrine molecules to reverse ECM remodeling to attenuate cardiac hypertrophy, and reached a theoretical heart infection rate of 1.67%. So that, it revealed a potential for administered AAV dose can be decreased by 10 times while still achieving the biological therapeutic effect for cardiac hypertrophy or fibrosis. The graphical abstract of our findings is presented in Fig. 1x.

## Supplementary information

Supplementary_Materials

Data S1

Data S2

Data S3

Data S4

English editing certification

## Data Availability

All the sequencing data has been deposited at GSA (CRA003779). Other data sets used and/or analyzed during the current study are available from the corresponding author on reasonable request.
