# Supplementary material for: Non-cell-autonomous manner of AAV administration to attenuate cardiomyocyte hypertrophy by targeting paracrine signaling on ECM to reduce viral dosage
Source: Signal Transduct Target Ther. 2022 Jan 3;7:2. doi: 10.1038/s41392-021-00715-z (PMC8724271; doi:10.1038/s41392-021-00715-z)
Supplement: Supplementary file 1 — Supplementary_Materials [file 41392_2021_715_MOESM1_ESM.docx]

Supplementary Materials for

**Non-cell-autonomous manner of AAV administration to attenuate cardiomyocyte hypertrophy by targeting paracrine signaling on ECM to reduce viral dosage**

Lei Liu^1^#, Peng Yue^1^#, Yue Zhang^1^#, Yimin Hua^1^#, Wenwei Bi^2^, Hualin Yan^3^, Hongyu Liao^1^, Jiawen Li^1^, Kaiyu Zhou^1^*, Yifei Li^1^*

1. Key Laboratory of Birth Defects and Related Diseases of Women and Children of MOE, Department of Pediatrics, West China Second University Hospital, Sichuan University, Chengdu, Sichuan 610041, China.

2. Institute of Neuroscience and Medicine 4, INM-4, Forschungszentrum Jülich, Jülich, Germany.

3. Department of Medical Ultrasound, West China Hospital, Sichuan University, Chengdu, Sichuan 610041, China.

*Correspondence:

Kaiyu Zhou: Email: kaiyuzhou313@163.com;

Yifei Li: Email: liyfwcsh@scu.edu.cn (leading contact)

**This PDF file includes:**

Figure S1 to S12

Table S1 to S2

Data S1 to S4

Materials and Methods

**Plasmids and AAV construction**

AAV-cTnT-U6-TuD-miR-133-GFP, AAV-cTnT-U6-pre-miR-133-GFP, AAV-cTnT-U6-GFP and AAV-cTnT-U6-Cre-GFP plasmids are constructed and validated by GeneCopoeia. AAV9-Rep/Cap and pHelper plasmids were available at Addgene. The sequences of AAV plasmids were listed in Supplementary Table 1.

The AAV production procedure was according to the previous study^1^. Plasmids (14 µg AAV- cTnT, 14 µg Rep/Cap, and 32µg pHelper) were transfected into15-cm plate of HEK293T cells using PEI transfection reagent (Polysciences, 23966-2). Cells were scraped off of plates after 2-3 days of transfection, and collected with cell culture medium. Resuspended the cell in lysis buffer (20 mM Tris, pH 8, 150 mM NaCl, 1 mM MgCl2, 50 µg/ml benzonase), lysed by three freeze-thaw cycles. Precipitated the cell culture medium by PEG 8000 (VWR, 97061-100), then resuspended in lysis buffer. The kit of AAV purification (TaKaRa, 6666) was used to purify AAV, and the procedure was as the instructions.

**Animal Experiments**

C57BLj mice were purchased from Nanjing model animal center. The AAC surgery was performed on 8-week-old mice and 8 for each group, which were anesthetized with chloralhydrate (4%, inhalation, 0.01ml/g). A longitudinal incision around 1cm of the skin of abdomen was made with scissors below the sternum. After the abdomen cavity was opened, abdominal intestine was taken out and wrapped with gauze soaked in warm saline. Isolated abdominal aorta, locating at the left of the inferior vena cava. Placed 0.26 mm ligation pole parallel to the abdominal aorta, and tied around the aorta and ligation pole using suture. Removed the ligation pole immediately to create a lumen with a fixed stenotic diameter. The abdominal cavity was closed, and the mice were given intramuscular injection of 10 to 20 thousand units of penicillin for three days to prevent infection.

Yap1^flox/flox^ mice were purchased from Jax Lab (027929). This kind of mice were used to generated tissue-specific *Yap1* knock-out mice using AAV-Cre-GFP after AAC surgery. And H11em1Cin^CAG-lsl-Yap1-P2A-mCherry^ mice were constructed by Nanjing Gembio Co., Ltd (T010798).

Yap1^flox/flox^ mice were PCR genotype using the primers listed as following:

Primer-F: AGGACAGCCAGGACTACACAG;

Primer-R: CACCAGCCTTTAAATTGAGAAC;

Yap1-overexpression mice were PCR genotyped using primers listed as following:

Primer-F: ATGCCCACCAAAGTCATCAGTGTAG;

Primer-R of AGGCGGGCCATTTACCGTAAGTTA.

Echocardiography was performed on a VisualSonics Vevo 2100 with Vevostrain software. Animals were wake during this procedure and held in a standard handgrip. Echocardiography was performed blinded to all groups.

To inject AAV into mice, the body weights of the animals were measured after anesthesia in an isoflurane chamber. The amount of AAV was calculated according to body weight. AAV-miR-133-inhibitor or AAV-GFP virus was subcutaneously injected as a dosage of 5×10^10^vg/g at birth day (P0) to mice. And intraabdominal injection of AAV-pre-miR-133 or control AAV-GFP virus with a reduced dosage of 2×10^10^vg/g at P28 of mice to establish a less infection rate at P28.

All procedures were performed following protocols approved by the West China Second University Hospital, Sichuan University, Animal Care and Use Committee.

**CM culture**

CMs were isolated using the Neomyt Kit (Cellutron, NC-6031). Cells from neonatal hearts of H11em1Cin^CAG-lsl-Yap1-P2A-mCherry^ mice were plated on 1% Matrigel (Corning, 354234)-coated plates in CM culture media (low glucose DMEM (Gibco), 5% horse serum (American Type Culture Collection, 30-2040), 2% chicken embryo extract (VWR, 100356-958)). The following day (day 1), adenovirus (Ad-TnT-Cre (Ad-Cre) or Ad-TnT-LacZ (Ad-LacZ)) was added at a multiplicity of infection of 10, which caused >90% cTNT+ cells to become mChery+. The following day, the medium was replaced with CM culture medium and subsequently changed daily.

**Histology and single CM isolation**

Hearts were harvested immediately after mice were euthanized by CO_2_, and the tissue was fixed by 4% paraformaldehyde overnight at 4 °C. Fixed cardiac tissues were cryoprotected by soaking in 30% sucrose for 2-4 hours at room temperature. The tissues were embedded in optimal cutting temperature (OCT) compound (SAKURA, Tissue-Tek). Six micrometers of cryo-sections were cut using a cryostat (Leica, CM3050). Masson staining (G1343-7, Solarbio, China) for the presence of interstitial collagen fiber accumulation was a marker of cardiac ECM remodeling. The ratio of interstitial fibrosis to the total left ventricular area was calculated from 15 randomly selected microscopic sidles in individual sections per heart using a camera attached to a Leica DM2000 microscope, with images further analyzed by ImageJ, excluding coronary vessels and perivascular regions.

Mice were anesthetized with isoflurane and the hearts of which were isolated and suspended onto a Langendorff perfusion system. First, pumped perfusion buffer (37 °C) into the heart through coronary artery to flush out blood. Second, digestion buffer including Collagenase II (Sigma, C-6885) was pumped into heart for 5 min to dissociate CMs. Then, removed the digested heart from the system and gently dissociated into single CMs in 10% fetal bovine serum (FBS)/perfusion buffer. Finally, removed undigested tissues through a 100 µm cell strainer. Concentrated the isolated CMs by 15 × g centrifugation for 5 min and resuspended the cell in culture medium (Dulbecco's modified Eagle's medium (Gibco), 10% FBS, pen/strep (Gibco), 10 µM blebbistatin). Cultured the CMs on laminin-coated coverslips for ~2 hours at 37 °C with 5% CO2 to allow cells to attach to the coverslips and prepared cells for immunofluorescence.

**Immunofluorescence and analysis**

CMs were fixed on coverslips by 4% paraformaldehyde for 15 min. The CMs and tissues were permeabilized by 0.1% Triton for 10 min, and blocked in 4% bovine serum albumin/PBS (blocking buffer) for 2-4 hour. The CMs and tissues were incubated with primary antibodies diluted in blocking buffer overnight at 4 °C (Anti-Wheat Germ Agglutinin antibody (Abcam, ab178444). The CMs and tissues were incubated with secondary antibodies (Abcam, ab150063) and DAPI dye (Abcam, ab150063) for 2 h (Abcam, ab104139).

Laser scanning confocal microscope (Olympus, FV1000) were taken to determine confocal fluorescence images. Fluorescence intensity and cell size were measured by ImageJ.

**RNA-seq and miRNA-seq**

Mice hearts were flash frozen in liquid nitrogen. 3 or 2 hearts were pooled for each replicate. RNA purity was checked using the NanoPhotometer^®^ spectrophotometer (IMPLEN, CA, USA). RNA concentration was measured using Qubit^®^ RNA Assay Kit in Qubit^®^ 2.0 Flurometer (Life Technologies, CA, USA). RNA integrity was assessed using the RNA Nano 6000 Assay Kit of the Bioanalyzer 2100 system (Agilent Technologies, CA, USA). Sequencing libraries were constructed using NEBNext^®^ UltraTM RNA Library Prep Kit for total RNAs and NEBNext^®^ Multiplex Small RNA Library Prep Set for miRs for Illumina^®^ (NEB, USA) and sequenced on an Illumina HiSeq 2000 (125 nt paired end for RNA-seq and 50 nt single end for miR-seq). The sequencing experiments had been done by Novogene Co., Ltd, Beijing, China. Transcript abundance was determined by Tophat alignment followed by HTSeq-Count and statistical analysis by DESeq2. Gene Ontology (GO) and Reactome enrichment analysis of differentially expressed genes were implemented by the clusterProfiler R package, in which gene length bias was corrected. GO and Reactome terms with corrected P-value less than 0.05 were considered significantly enriched by differential expressed genes. Differential expression analysis of miRNAs was performed using the DESeq R package (1.8.3). The P-values was adjusted using the Benjamini & Hochberg method. MiRNAs predicted targets were performed on TargetScan version7.1 (www.targetscan.org/vert). GO enrichment analysis of miRNAs was used on the target gene candidates of differentially expressed miRNAs. All the sequencing data has been deposited at GSA (CRA003779).

**Gene set enrichment analysis (GSEA).**

GSEA was performed on expressed genes according to the software manual. Gene sets with a nominal p value of <0.05 and an FDR of <0.25 were considered significant. All expressed genes were Log2 or Log10 transformed, centered, and unsupervised hierarchical clustering was performed using the k-mean clustering method with Cluster 3.0 software. R software was used to visualize the clustered heatmaps. Gene sets displayed by MsigBD V7.2. Gene set of miR-133 targeting genes was generated with all the predicted binding genes of miR-133 using TargetScan. Gene sets of embryonic and adult heart genes were generated according to the gene list by Uosaki et al. 2015^2^.

**Reverse transcription-quantitative PCR analysis and ELISA assay**

NucleoZOL Kit (740404.200) was used to isolate small and large RNA in two separate fractions. Genomic DNA removal and reverse transcription for large RNA was performed using PrimeScript™ RT reagent Kit with gDNA Eraser (Takara, RR047A). Real-time PCR for large RNA was performed using a Biorad CFX96 with TB Green Advantage qPCR Premix User Manual (Takara, 639676), and qPCR primers were listed as in Supplementary Table 2.

All-in-One^TM^ miRNA qRT-PCR Detection Kit For quantitative detection of mature miRNA (GeneCopoeia, AOMD-Q020) was used for reverse transcription and real-time PCR analysis of small RNA. The primers for miR-133 or U6 were provided by the GeneCopoeia.

The CTGF levels were also quantitatively determined in triplicate with ELISA kit (Mskbio, kt58712) following the manufacturer’s protocols.

**Luciferase reporter assays**

Putative binding sites located in the 3′UTR of *Ctgf* were identified using TargetScan version7.1 ([www.targetscan.org/vert)](http://www.targetscan.org/vert)). Plasmids PEZx-FR02 (Genecopoeia Co, Ltd) carrying the luciferase gene linked to a fragment of the *Ctgf* 3’-UTR harbouring miR-133 putative binding sites were constructed, named as ctgf-3’UTR-wt. A mutant 3’-UTR of *ctgf* was constructed by mutating AUUUGUUGAGAGUGUGACCAAAA into AUUUGUUGAGAGUGUCTGGTTTA, named as ctgf-3’UTR-Mut. For luciferase reporter assay, H293T were seeded in 96-well plates overnight and then co-transfected with Wt or Mut luciferase reporter vector and miR-133, miR-NC using Lipofectamine 3000 transfection reagent. Dual-Luciferase® Reporter Assay System kit (Promega, E1910) was used for luciferase activity assay in each group after 48 h post-transfection.

**Affecting model establishment by Python**

In the python codes of “voxels_2D.py”, the four corners of the initial cell, with its cross-section area being 20*20 square block, are the starting points of cell coverage, meaningly, four centers of the coverage circle, each circle has been presented as four quarter arcs with different radius across different layers.

For the middle/plane layer, the covering radius is 84.5. Since cell length is 120, the full coverage of layer on the top or at the bottom is impossible, only covering half of the cell length 60 is possible, while for top or bottom layer, the covering radius in this case is the square root of 84.5^2^- (cell length/2) ^2^.

Python codes were uploaded as Supplementary files 1 &2.

**Statistical analysis**

The data are expressed as the mean ± standard deviation. Two-way analysis of variance was used to assess the statistical significance of the differences between groups followed by Student-Neuman-Keuls test for multiple comparisons. P<0.05 was considered to indicate a statistically significant difference. Statistical analysis was performed using SPSS software version 21.0 (IBM Corporation, Armonk, NY, USA).

**Reference:**

1 Guo, Y. *et al.* Hierarchical and stage-specific regulation of murine cardiomyocyte maturation by serum response factor. *Nature communications* **9**, 3837, doi:10.1038/s41467-018-06347-2 (2018).

2 Uosaki, H. *et al.* Transcriptional Landscape of Cardiomyocyte Maturation. *Cell reports* **13**, 1705-1716, doi:10.1016/j.celrep.2015.10.032 (2015).

**
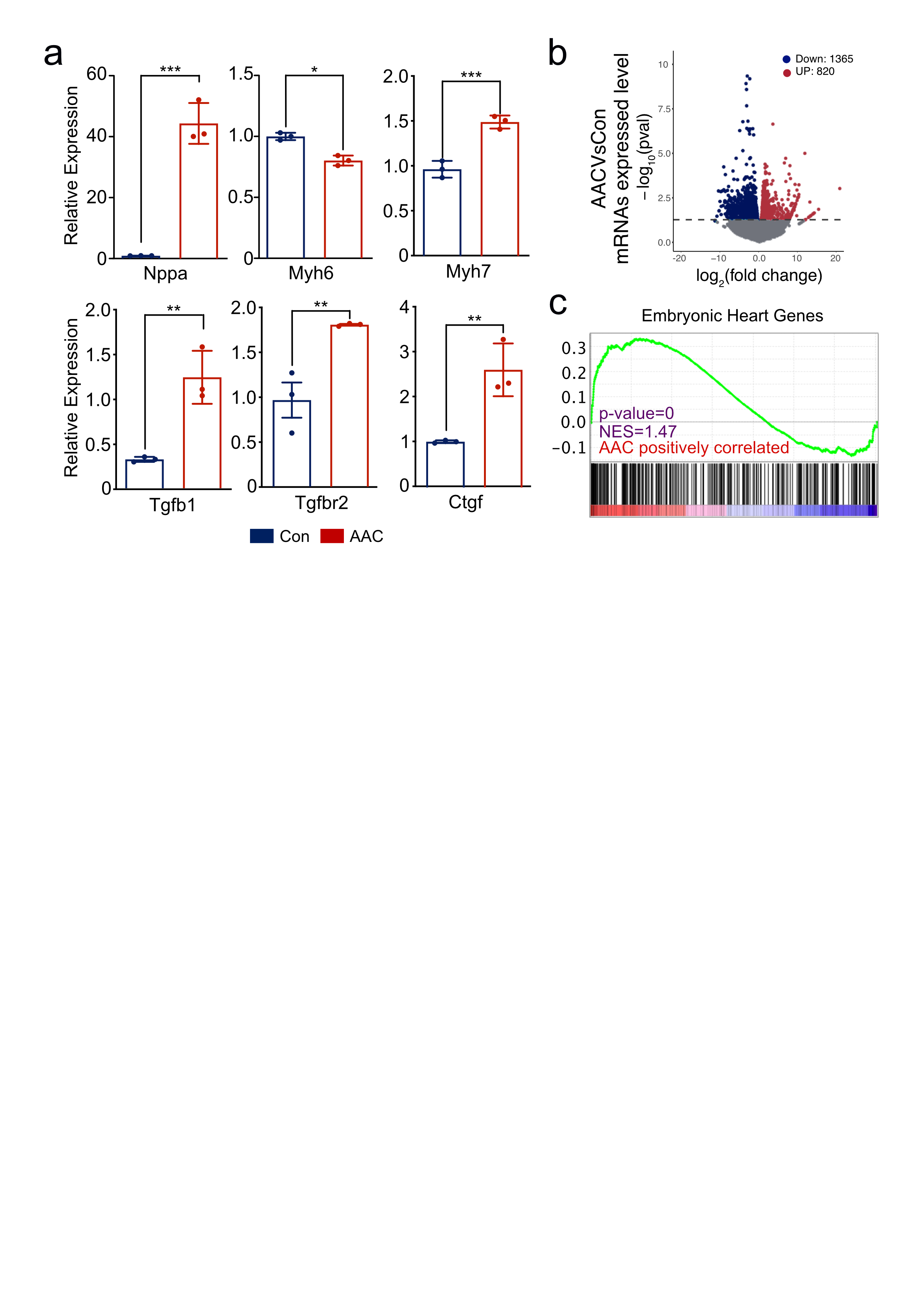
**

**Figure. S1**. **a.** The hypertrophic gene *Nppa* and *Myh7* dramatically increased in AAC hearts by qPCR. Fibrotic genes *Tgfb1*, *Tgfbr2* and *Ctgf* were also elevated in AAC hearts. N = 3 biologically independent samples. **b.** Volcano plot showing the log fold changes and log p-value of each gene of RNA-seq in AAC hearts. **c.** GSEA revealed enrichment of embryonic heart genes in AAC hearts. All the data were shown as mean ± SD, *P < 0.05; **P < 0.01; ***P < 0.001. AAC, abdominal aorta contraction; NES, normalized enrichment score.


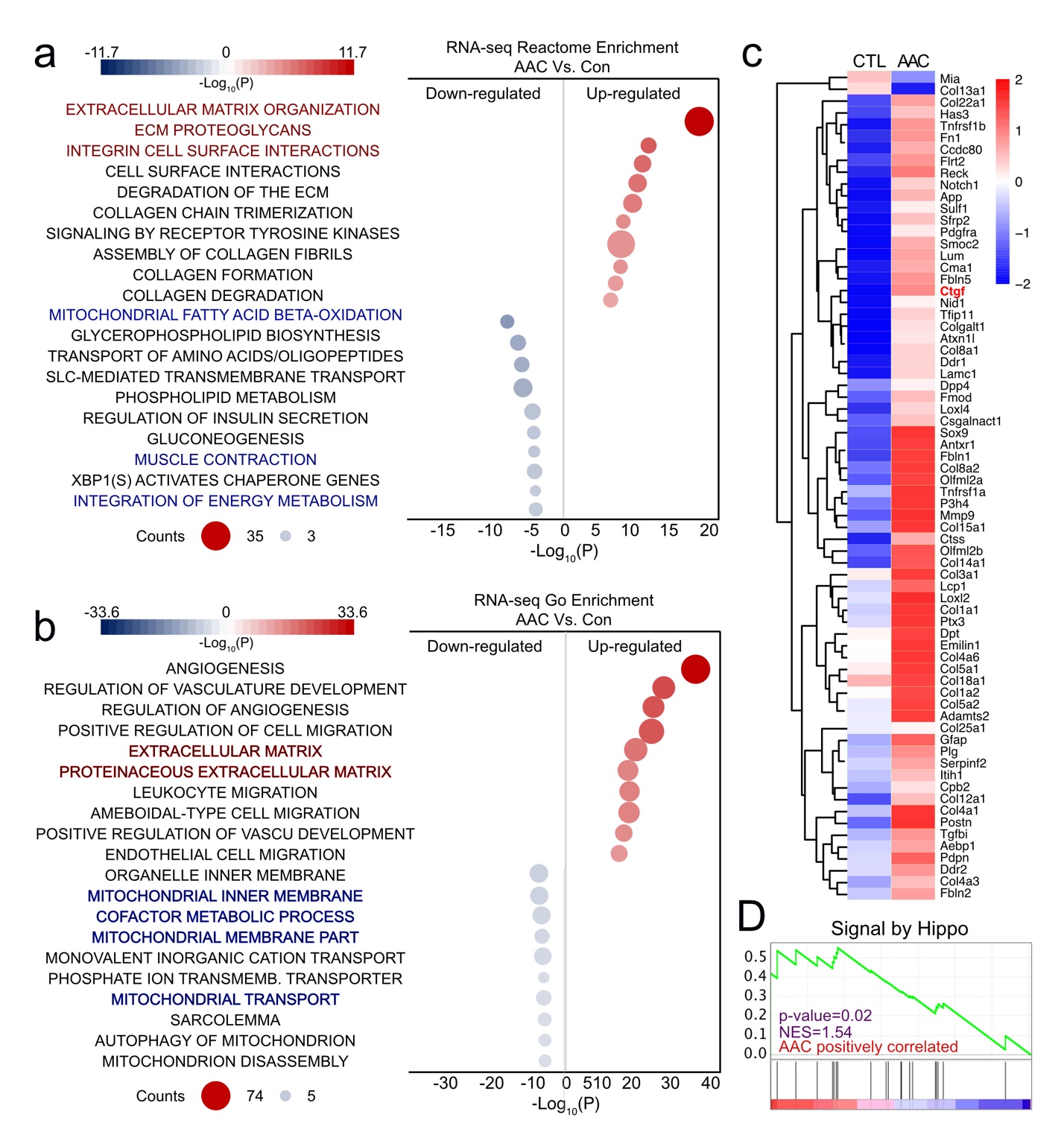


Figure. S2. RNA-seq for AAC mice and control ones. a-b. The enrichment terms analyses confirmed the findings at activation of ECM remodeling and mitochondrial dysfunction related pathways. a for Reactome enrichment and b for Go terms. as the terms of ECM component, proteinaceous ECM and ECM had been targeted. c. Heat-map on ECM remodeling related genes. d. GSEA showed Hippo signal activation in AAC hearts. AAC, abdominal aorta contraction; NES, normalized enrichment score.


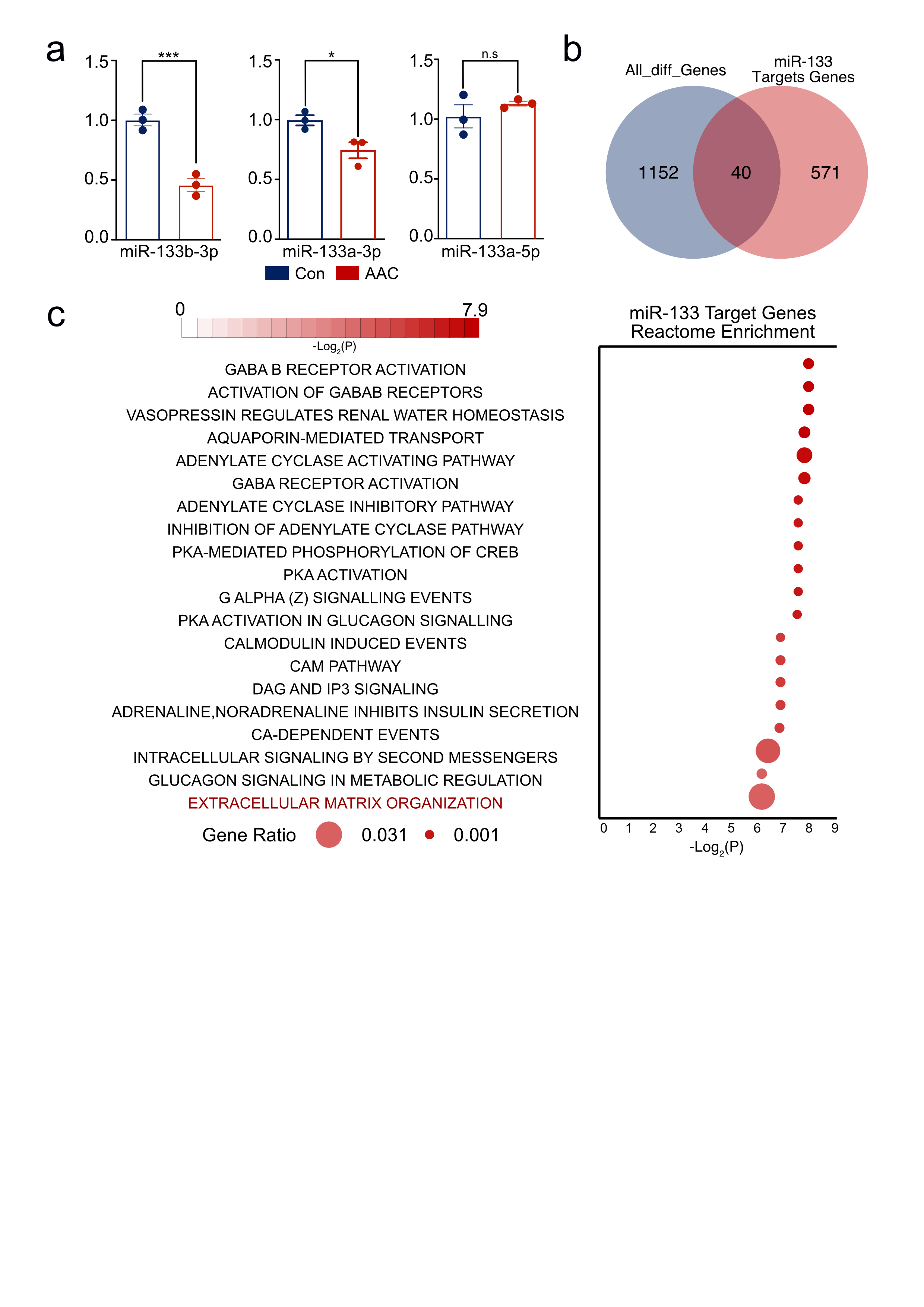


**Figure. S3.** miR-133 was predicted targeting ECM. **a.** qPCR for miR-133 related miRs showed a reduction of relative expression to control ones in TAC treated mice. **b.** Venn map present the co-identified genes of all different expressed genes and miR-133 binding ones. **c.** The Reactome terms analysis for 40 co-appeared genes revealed the miR-133 involved in the regulation of ECM organization in hypertrophic process. All the data were shown as mean ± SD, *P < 0.05; ***P < 0.001. AAC, abdominal aorta contraction.

**
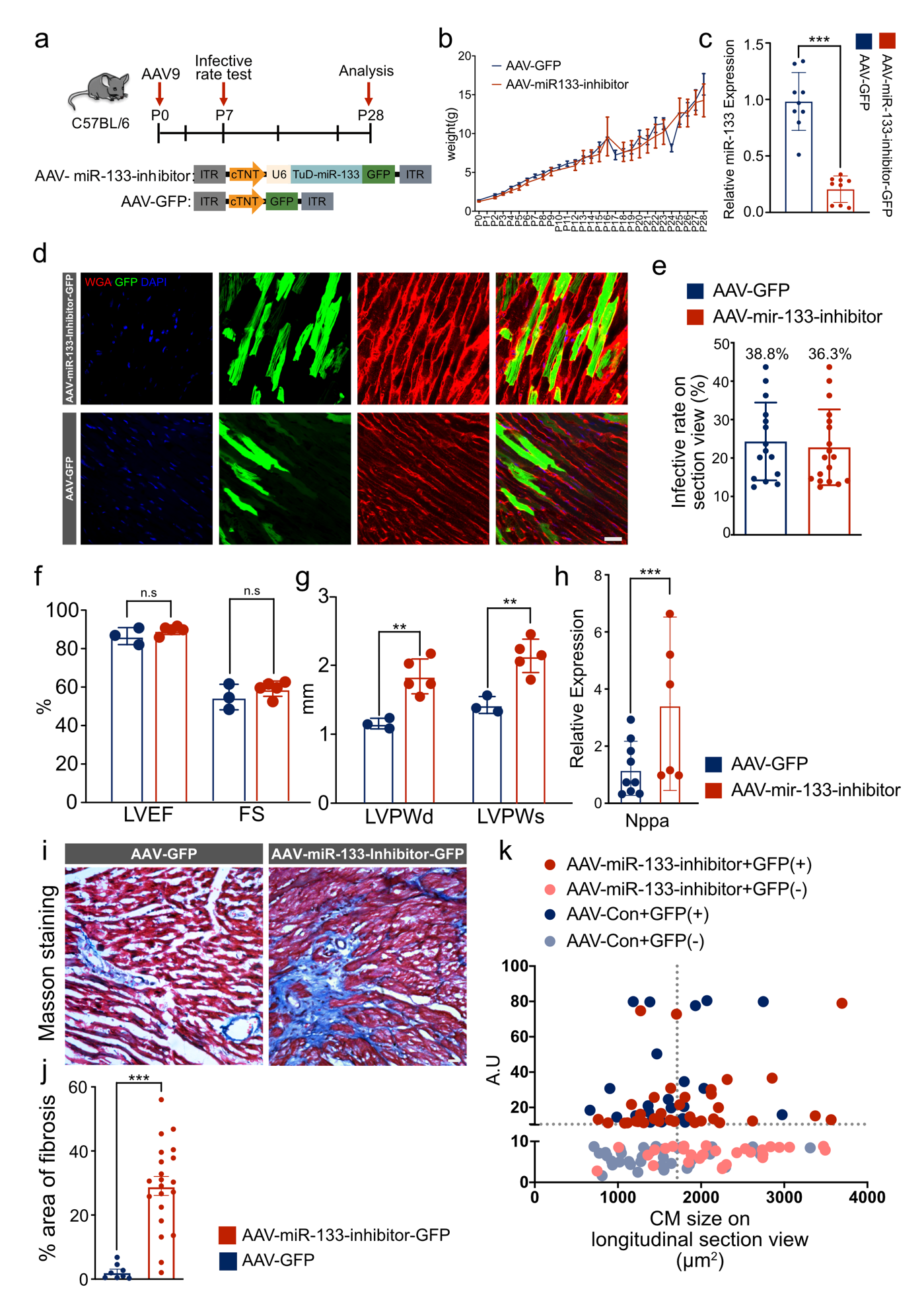
**

**Figure. S4.** Inhibition of miR-133 induced hypertrophic phenotype of heart. **a.** Workflow of miR-133 inhibition using AAV. **b.** The changes of weight from P0 to P28 (N = 8 biologically independent samples). **c.** qPCR showed miR-133 inhibited significantly. **d-e.** The AAV infected rates were calculated at P7 by WGA staining by accounting GFP+ CMs (N = 3 biologically independent samples). **f-g.** The echocardiographic results of AAV-miR-133-inhibitor injected mice, demonstrating the thickness of LVPW in both systolic and diastolic periods (N = 4 biologically independent samples). **h.** The relative mRNA level of the hypertrophic gene of *Nppa* in the hearts which were subjected to AAV induced miR-133 inhibition (N = 4 biologically independent samples). **i-j.** Masson staining demonstrated the fibrotic area significantly observed in miR-133 inhibited hearts, which showed a 28% percentage area had been positive for collagen staining. **k.** Scatter plots for individual CMs size between AAV-miR-133-inhibitor and AAV-Con groups with or without GFP expression. Scale Bar, 50 μm; All the data were shown as mean ± SD, **P < 0.01; ***P < 0.001. AAC, abdominal aorta contraction; AAV, adeno-associated virus; A.U, CM, cardiomyocyte; ECM, extracellular matrix; FS, fraction shortening; LVEF, left ventricle ejection faction; LVPWs, left ventricular partial wall, systolic; LVPWd, left ventricular partial wall, diastolic.

**
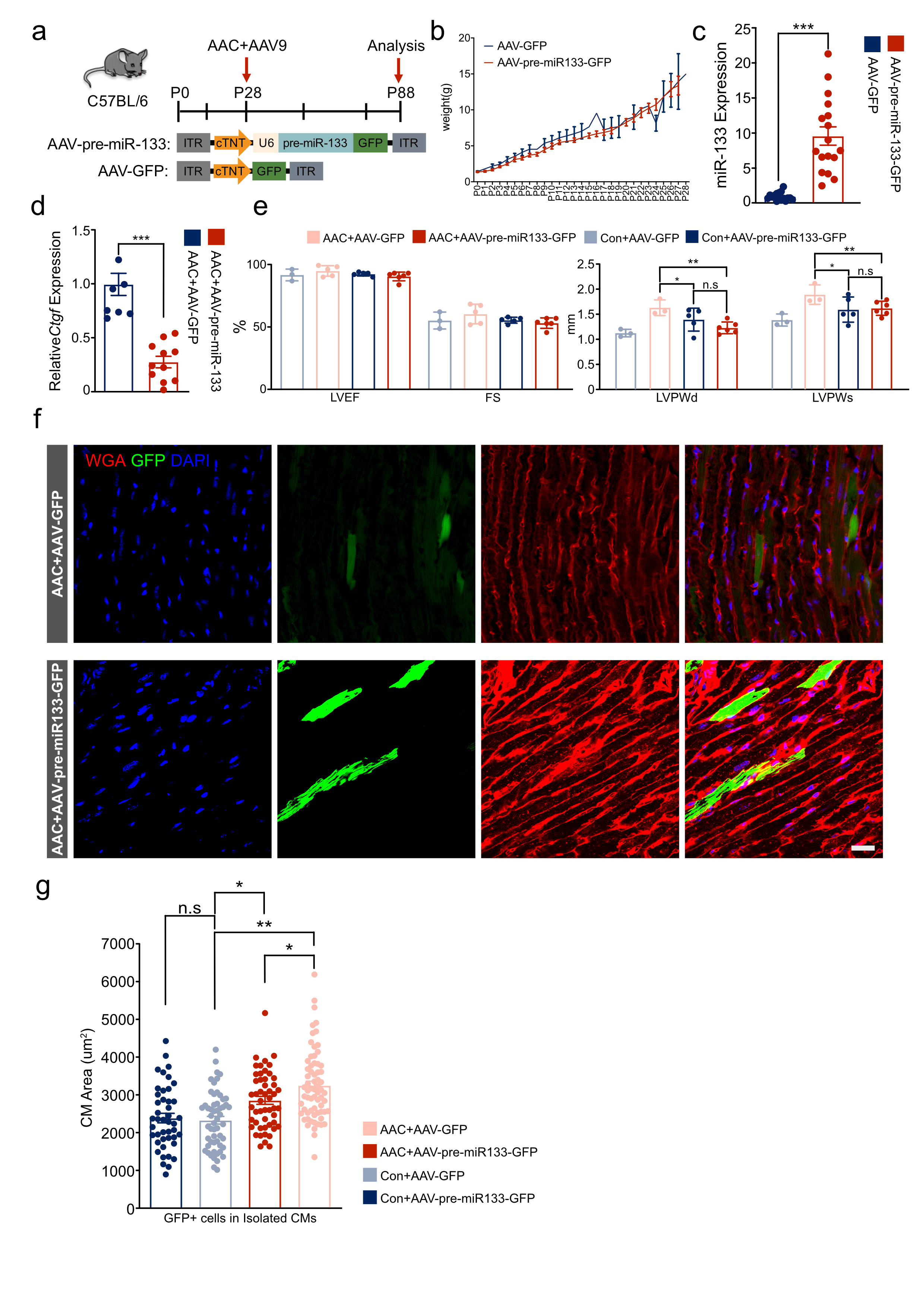
**

**Figure. S5.** AAV delivering miR-133 attenuated the cardiac hypertrophy induced by AAC. **a.** Workflow for delivering miR-133 using AAV to mice subjected AAC. **b.** The changes of weight from P0 to P28 (N = 8 biologically independent samples). **c.** qPCR measured the expression level of miR-133 in hearts after injected AAV-pre-miR-133 (N = 4 biologically independent samples). **d.** The expression of *Ctgf* dropped significantly after AAV-miR-133 administration (N = 4 biologically independent samples). **e.** The echocardiographic results of AAV-pre-miR-133 injected hearts, demonstrating the thinner of LVPW in both systolic and diastolic periods (N = 4 biologically independent samples). **f-g.** The WGA staining results of heart tissues on longitudinal view (N = 4 biologically independent samples), and the cell size was measured in 10 fields/slice in both groups. (n > 20 CMs per individual heart). Scale Bar, 50 μm; All the data were shown as mean ± SD, *P < 0.05; **P < 0.01; ***P < 0.001. AAC, abdominal aorta contraction; AAV, adeno-associated virus; CM, cardiomyocyte; FS, fraction shortening; LVEF, left ventricle ejection faction; LVPWs, left ventricular partial wall, systolic; LVPWd, left ventricular partial wall, diastolic.

**
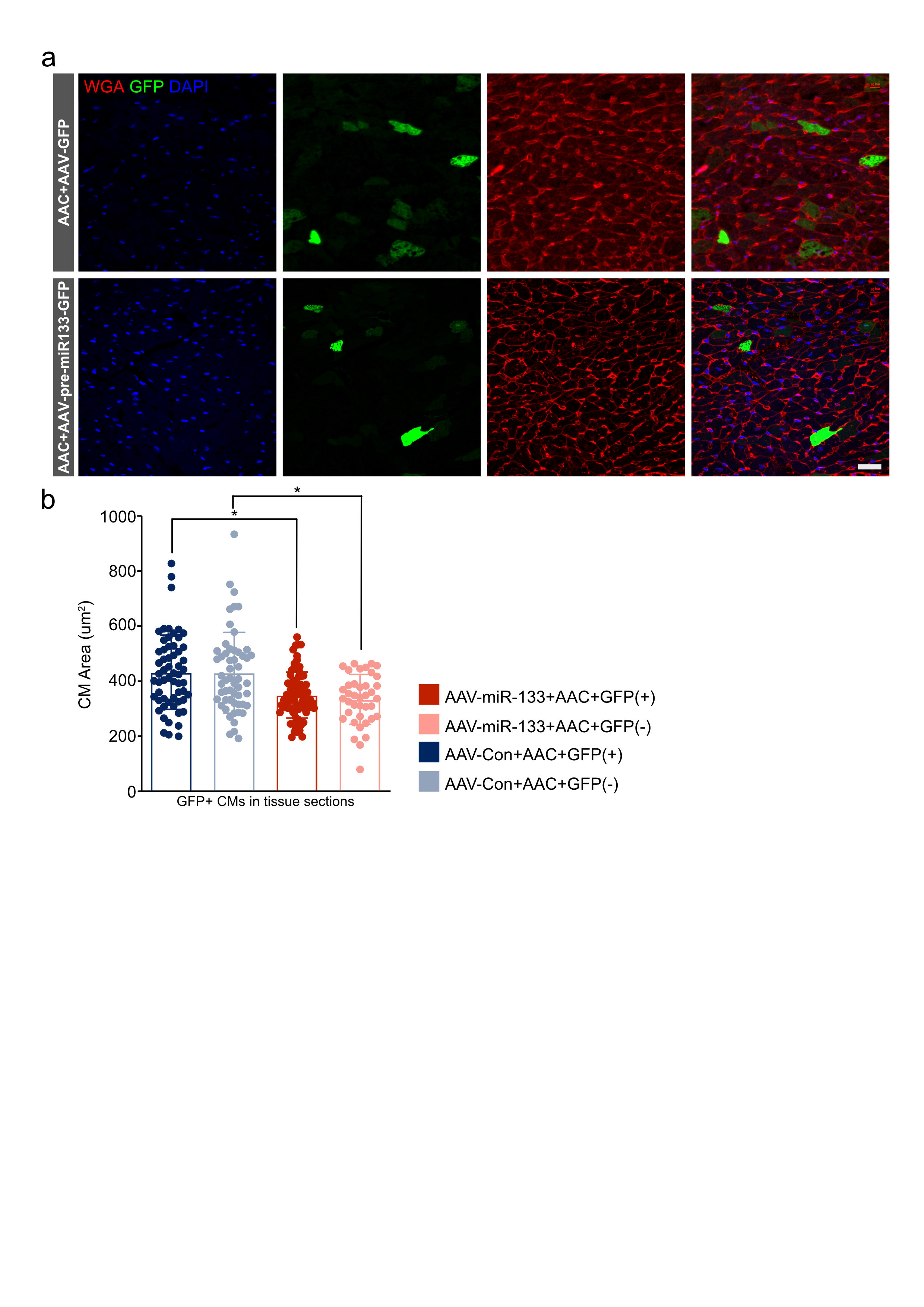
**

**Figure. S6.** The WGA staining results of heart tissues on transverse view (N = 4 biologically independent samples), and the cell size was measured in 10 fields/slice in both groups. (n > 20 CMs per individual heart). Scale Bar, 50 μm; All the data were shown as mean ± SD, *P < 0.05. AAC, abdominal aorta contraction; AAV, adeno-associated virus; CM, cardiomyocyte.

**
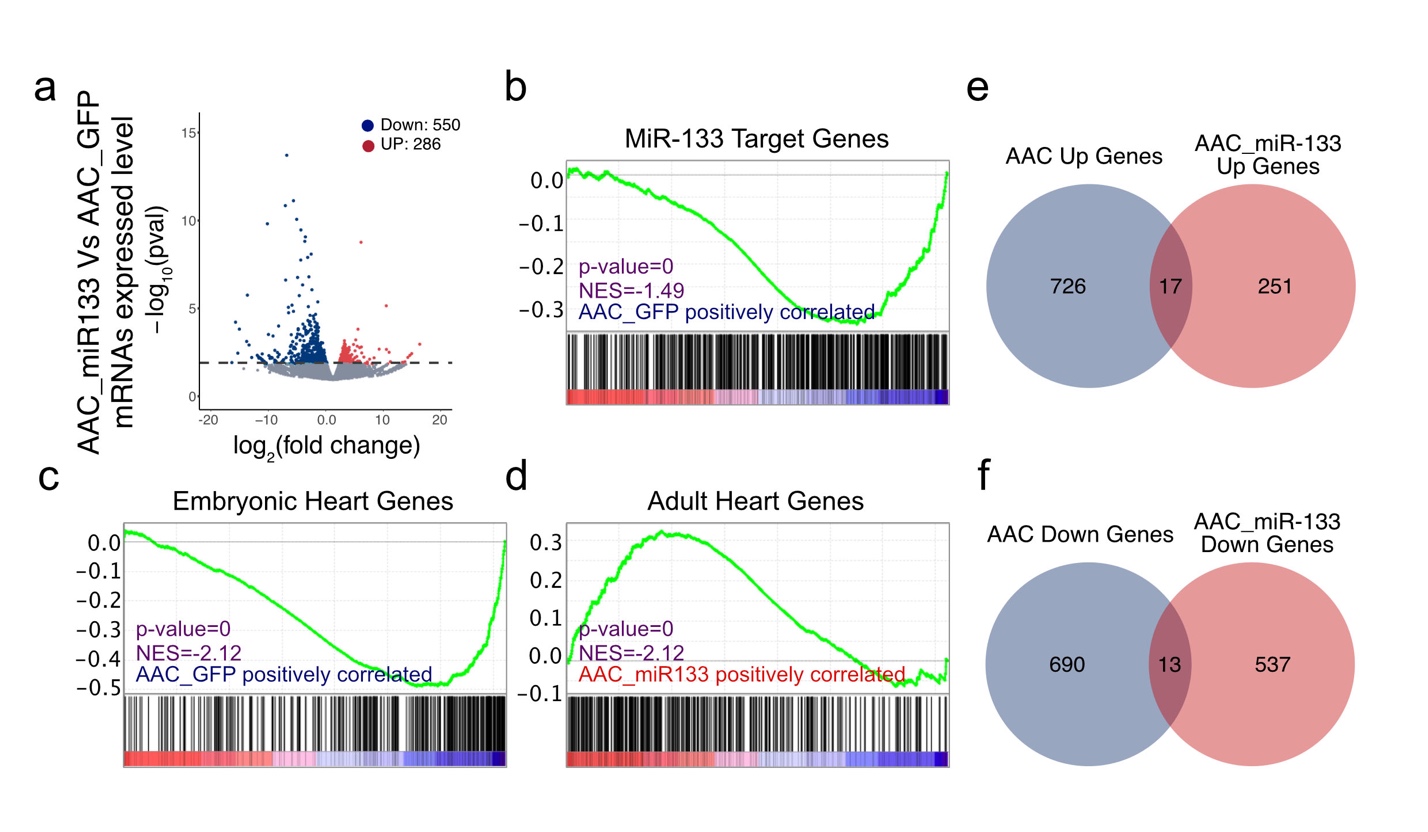
**

**Figure. S7.** RNA-seq analysis of hearts after AAV-miR-133 administration. **a.** Volcano plot showing the log fold changes and log p-value of each gene of RNA-seq in AAV-miR-133 subjected AAC hearts. **b-d.** GSEA revealed enrichment of adult heart genes, while dysregulations were recorded of miR-133 targeting genes and embryonic heart genes in AAC hearts after AAV delivering miR-133. **e-f.** There were few of sharing genes between AAC hearts before and after delivering miR-133 both in up and down regulated ones. AAC, abdominal aorta contraction; NES, normalized enrichment score.

**
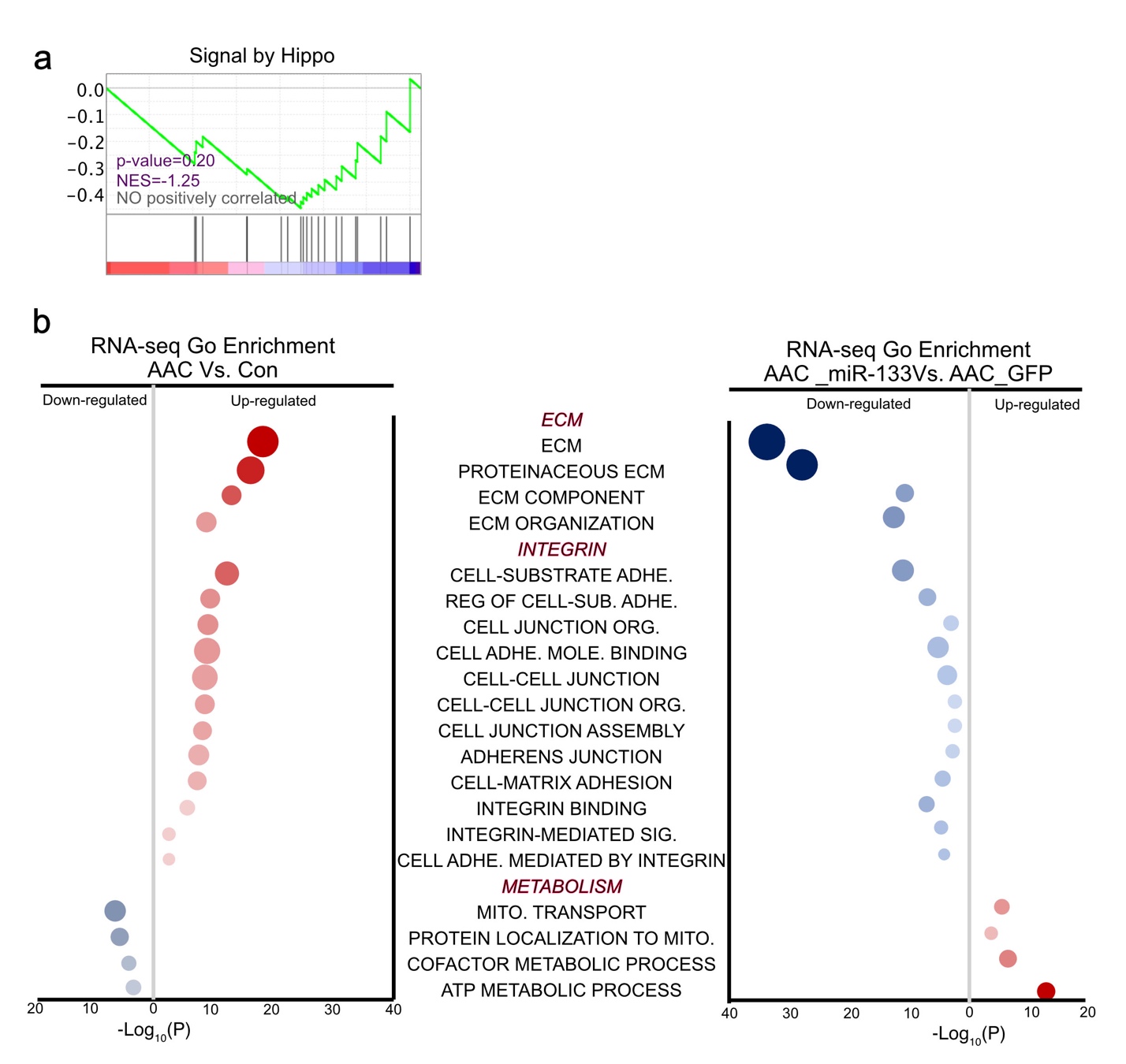
**

**Figure. S8.** Comparison on gene sets enrichment between AAC hearts before and after delivering miR-133. **a.** GSEA revealed no enrichment of Hippo signal in AAC hearts after AAV delivering miR-133. **b.** Scatter plots demonstrated the down-regulation of both ECM and integrin related pathways in AAC hearts with miR-133 delivering, while mitochondrial function related pathways were up-regulated. AAC, abdominal aorta contraction; NES, normalized enrichment score.

**
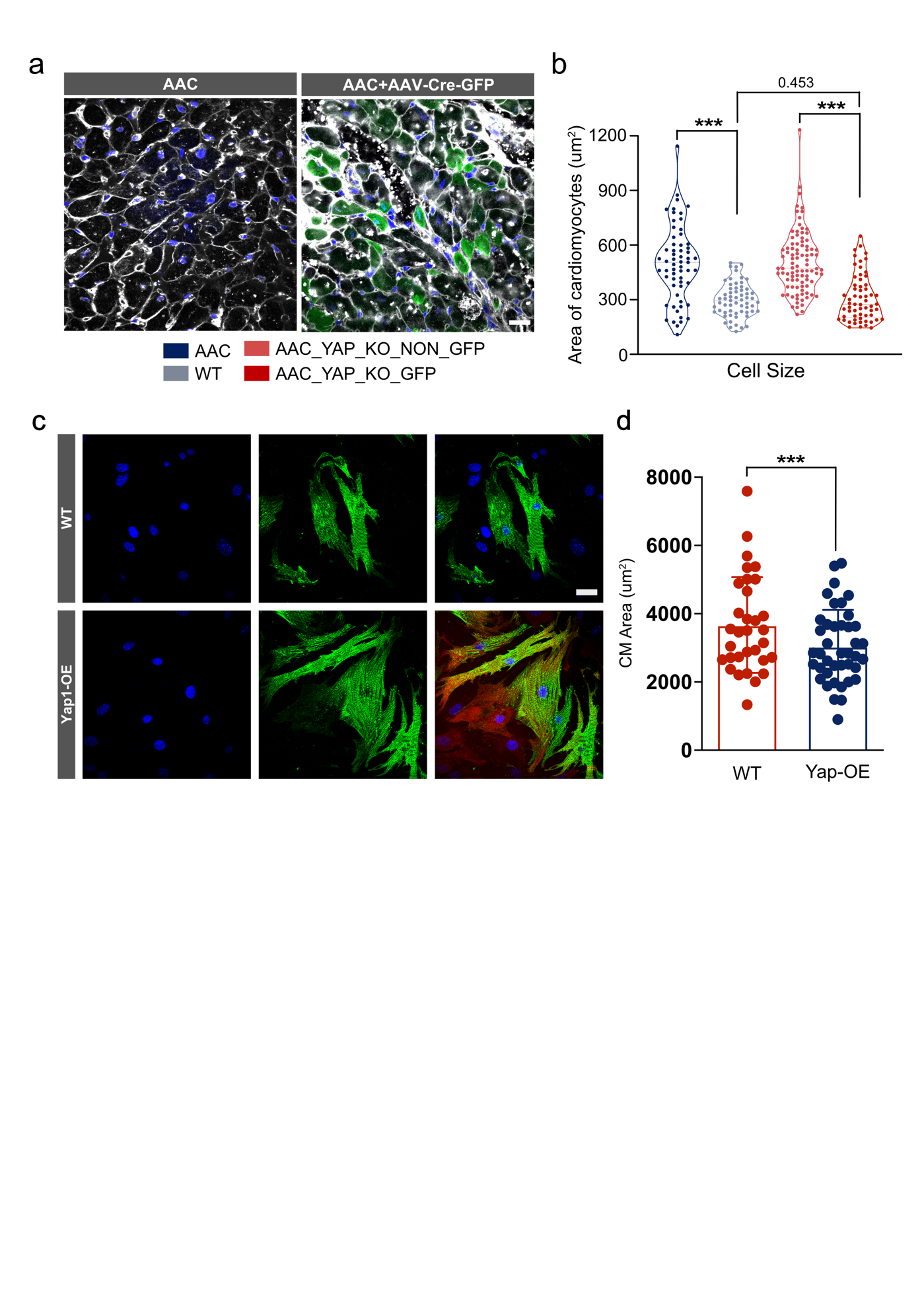
**

**Figure. S9.** Morphological changes of CMs after Yap1 knock out or overexpression. **a-b.** Yap1^flox/flox^ mice were subjected to AAC surgery, and AAV-cTnT-Cre-GFP was administrated to generate tissue specific *Yap1* knock out. And attenuation of hypertrophy could only be observed in Yap1-KO CMs (N = 4 biologically independent samples, n > 20 CMs per individual sample). **c-d.** Primary CMs culture with Ad-Cre administration had been performed and increased cell size had been observed (N = 4 biologically independent samples, n > 20 CMs per individual sample). Scale Bar, 50 μm; All the data were shown as mean ± SD, **P < 0.01. AAC, abdominal aorta contraction; AAV, adeno-associated virus; CM, cardiomyocyte; WT, wildtype; Yap1-OE, Yap1 overexpression.

**
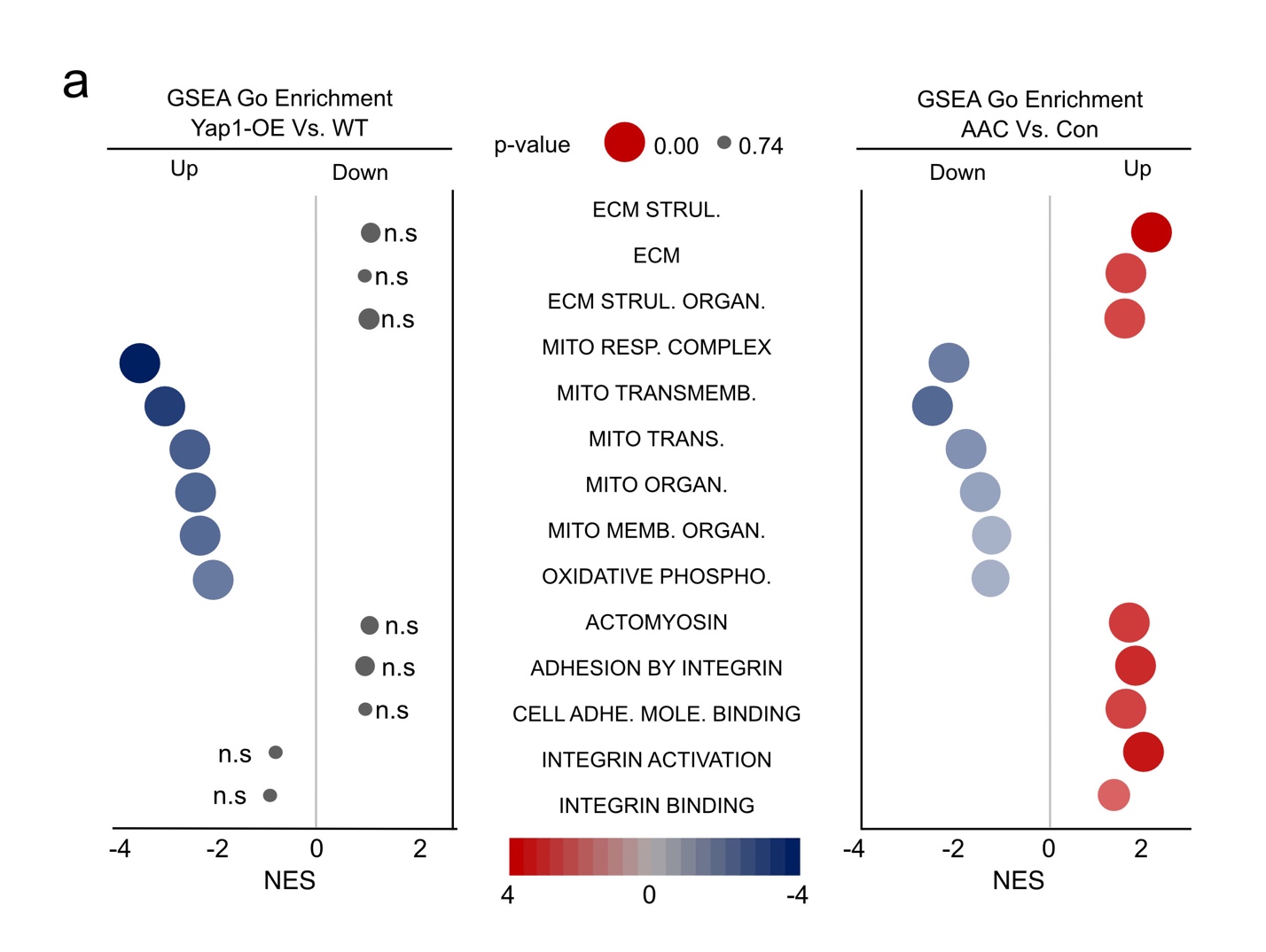
**

**Figure. S10.** Scatter plots of GSEA with MSigDB Hallmark gene sets (v 7.2) displaying ECM, mitochondrial function and integrin activation regulated pathways of between AAC hearts and Yap1-overexpression was induced using H11em1Cin^CAG-lsl-Yap1-mCherry^ CMs primary culture wirh Ad-cre administration. Results demonstrated mitochondrial dysfunction both enriched in both groups, while no differentiated displaying ECM and integrin related pathways enriched in Yap1-overexpression CMs. AAC, abdominal aorta contraction; AAV, adeno-associated virus; CM, cardiomyocyte; WT, wildtype; Yap1-OE, Yap1 overexpression.

**
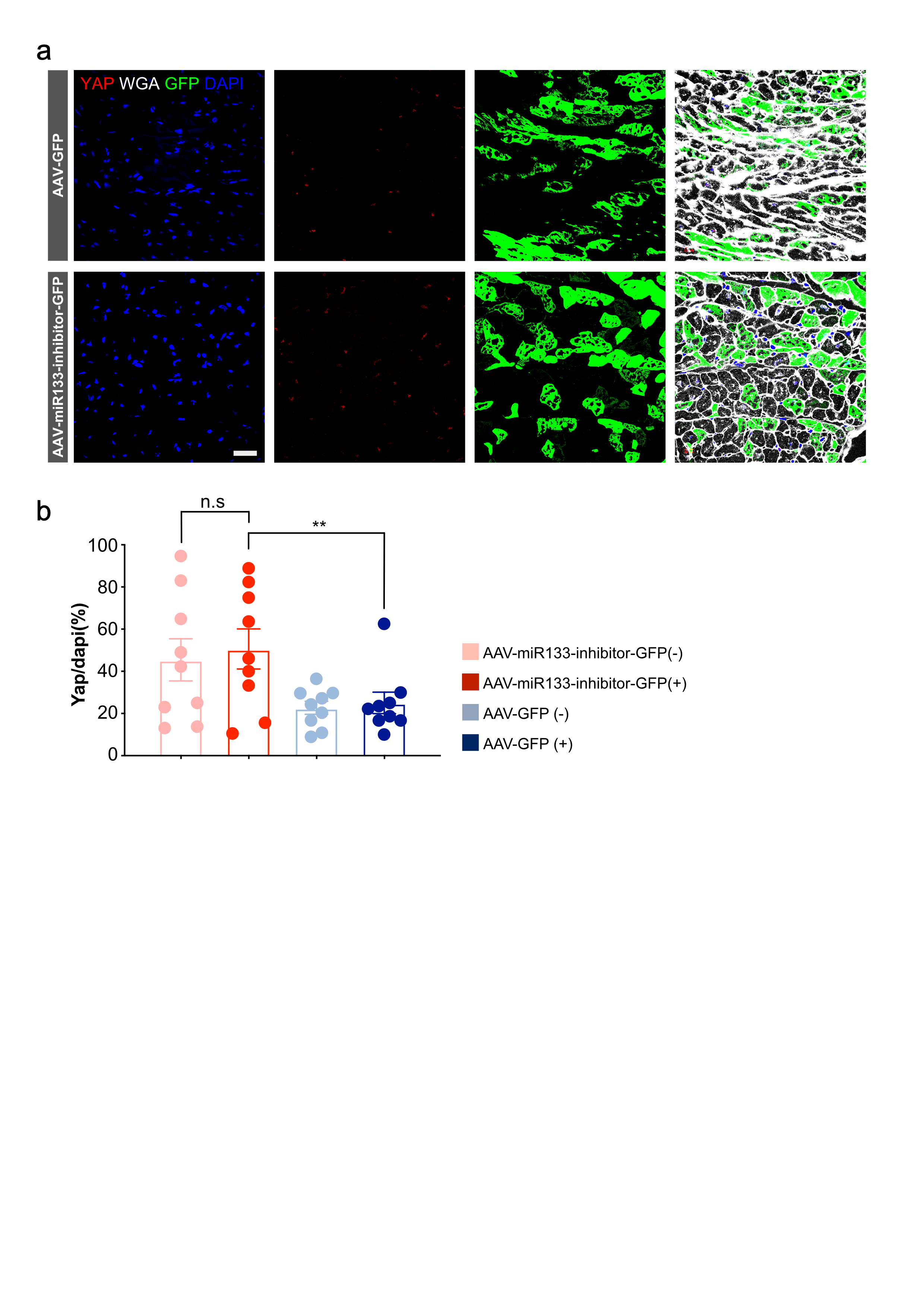
**

**Figure. S11.** YAP was activated in both infected and non-infected CMs after AAV-miR-133-inhibitor administration (N = 4 biologically independent samples). Scale Bar, 50 μm; All the data were shown as mean ± SD, **P < 0.01. AAV, adeno-associated virus.

**
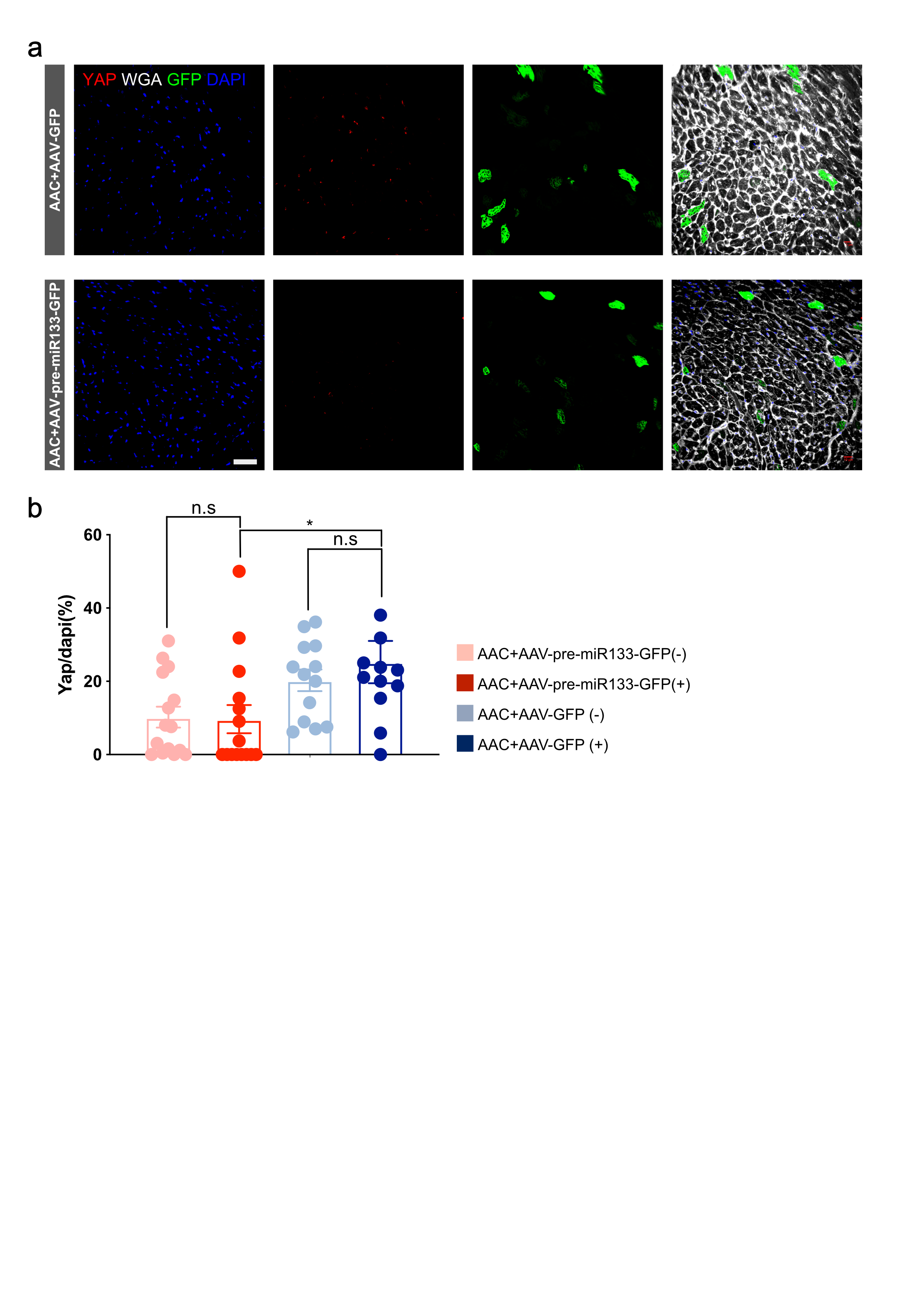
**

**Figure. S12.** YAP activation had been attenuated in both infected and non-infected CMs under exogenous miR-133 delivering to AAC hearts (N = 4 biologically independent samples). Scale Bar, 50 μm; All the data were shown as mean ± SD, *P < 0.05. AAV, adeno-associated virus.

**
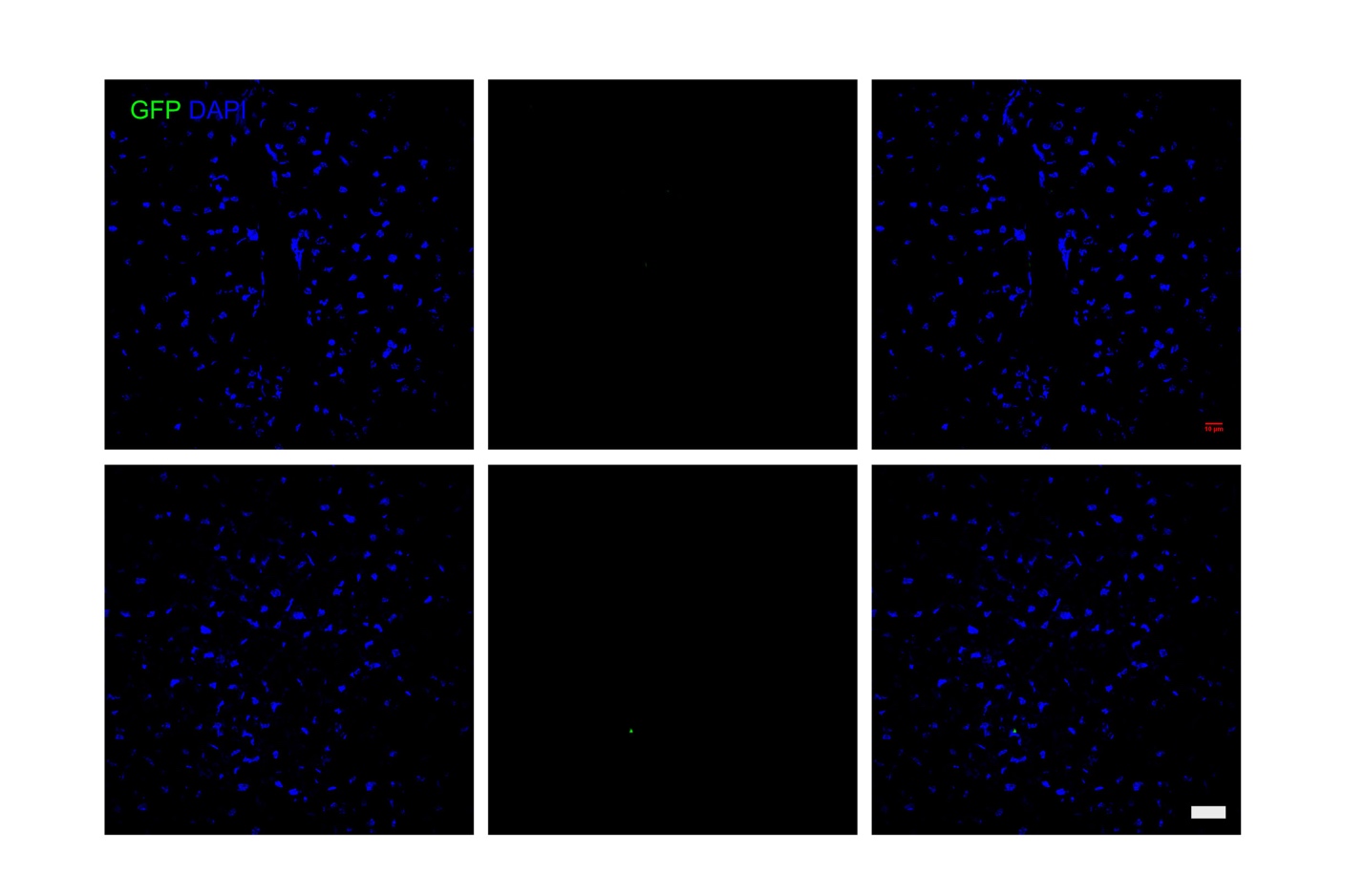
**

**Figure. S13.** YAP activation had been attenuated in both infected and non-infected CMs under exogenous miR-133 delivering to AAC hearts (N = 4 biologically independent samples). Scale Bar, 50 μm; All the data were shown as mean ± SD, *P < 0.05. AAV, adeno-associated virus.

**Table S1. The sequences of used AAV plasmid.**

| **AAV plasmid** | **the sequence of AAV plasmid** |
| --- | --- |
| **AAV-pre-miR-133** | CTGCGCGCTCGCTCGCTCACTGAGGCCGCCCGGGCAAAGCCCGGGCGTCGGGCGACCTTTGGTCGCCCGGCCTCAGTGAGCGAGCGAGCGCGCAGAGAGGGAGTGGCCAACTCCATCACTAGGGGTTCCTTGTAGTTAATGATTAACCCGCCATGCTACTTATCTACCAGGGTAATGGGGATCCTCTAGAACTATAGCTAGAATTCGCCCTTACGGGCCCATACAATTGGAGGGCCTATTTCCCATGATTCCTTCATATTTGCATATACGATACAAGGCTGTTAGAGAGATAATTGGAATTAATTTGACTGTAAACACAAAGATATTAGTACAAAATACGTGACGTAGAAAGTAATAATTTCTTGGGTAGTTTGCAGTTTTAAAATTATGTTTTAAAATGGACTATCATATGCTTACCGTAACTTGAAAGTATTTCGATTTCTTGGCTTTATATATCTTGTGGAAAGGACGAAACACCGACCGGTctgtttaacaactggtacactagtgTGGGAACCTCTAATACCTGTCATGCTATATTTCTACAAAAGAGCATTTAACCTGTTTGGATCCTACACCAGCAGCGGCAATGCTTTGCTAAAGCTGGTAAAATGGAACCAAATCGCCTCTTCAATGGATTTGGTCCCCTTCAACCAGCTGTAGCTATGCATTGATTACACATGGACAGCCAGTGTTTTATGAgcatgaattacatctcaacttactcaaCGGACCGCTTTTTTGGGCCCCCCCTCGAGGTCGGGATAAAAGCAGTCTGGGCTTTCACATGACAGCATCTGGGGCTGCGGCAGAGGGTCGGGTCCGAAGCGCTGCCTTATCAGCGTCCCCAGCCCTGGGAGGTGACAGCTGGCTGGCTTGTGTCAGCCCCTCGGGCACTCACGTATCTCCGTCCGACGGGTTTAAAATAGCAAAACTCTGAGGCCACACAATAGCTTGGGCTTATATGGGCTCCTGTGGGGGAAGGGGGAGCACGGAGGGGGCCGGGGCCGCTGCTGCCAAAATAGCAGCTCACAAGTGTTGCATTCCTCTCTGGGCGCCGGGCACATTCCTGCTGGCTCTGCCCGCCCCGGGGTGGGCGCCGGGGGGACCTTAAAGCCTCTGCCCCCCAAGGAGCCCTTCCCAGACAGCCGCCGGCACCCACCGCTCCGTGGGACGATCCCCGAAGCTCTAGAGCTTTATTGCGGTAGTTTATCACAGTTAAATTGCTAACGCAGTCAGTGCTTCTGACACAACAGTCTCGAACTTAAGCTGCAGAAGTTGGTCGTGAGGCACTGGGCAGGTAAGTATCAAGGTTACAAGACAGGTTTAAGGAGACCAATAGAAACTGGGCTTGTCGAGACAGAGAAGACTCTTGCGTTTCTGATAGGCACCTATTGGTCTTACTGACATCCACTTTGCCTTTCTCTCCACAGGTGTCCACTCCCAGTTCAATTACAGCTCTTAAGGCTAGAGTACTTAATACGACTCACTATAGGCTAGCCTCGAGAATTCACGCGGGCCGCCATGGTGAGCAAGGGCGAGGAGCTGTTCACCGGGGTGGTGCCCATCCTGGTCGAGCTGGACGGCGACGTAAACGGCCACAAGTTCAGCGTGTCCGGCGAGGGCGAGGGCGATGCCACCTACGGCAAGCTGACCCTGAAGTTCATCTGCACCACCGGCAAGCTGCCCGTGCCCTGGCCCACCCTCGTGACCACCCTGACCTACGGCGTGCAGTGCTTCAGCCGCTACCCCGACCACATGAAGCAGCACGACTTCTTCAAGTCCGCCATGCCCGAAGGCTACGTCCAGGAGCGCACCATCTTCTTCAAGGACGACGGCAACTACAAGACCCGCGCCGAGGTGAAGTTCGAGGGCGACACCCTGGTGAACCGCATCGAGCTGAAGGGCATCGACTTCAAGGAGGACGGCAACATCCTGGGGCACAAGCTGGAGTACAACTACAACAGCCACAACGTCTATATCATGGCCGACAAGCAGAAGAACGGCATCAAGGTGAACTTCAAGATCCGCCACAACATCGAGGACGGCAGCGTGCAGCTCGCCGACCACTACCAGCAGAACACCCCCATCGGCGACGGCCCCGTGCTGCTGCCCGACAACCACTACCTGAGCACCCAGTCCGCCCTGAGCAAAGACCCCAACGAGAAGCGCGATCACATGGTCCTGCTGGAGTTCGTGACCGCCGCCGGGATCACTCTCGGCATGGACGAGCTGTACAAGTAATAAGCTCGCGTGGTACCTCTAGAGTCGACCCGGGCGGCCTCGAGGACGGGGTGAACTACGCCTGAGGATCCGATCTTTTTCCCTCTGCCAAAAATTATGGGGACATCATGAAGCCCCTTGAGCATCTGACTTCTGGCTAATAAAGGAAATTTATTTTCATTGCAATAGTGTGTTGGAATTTTTTGTGTCTCTCACTCGGAAGCAATTCGTTGATCTGAATTTCGACCACCCATAATACCCATTACCCTGGTAGATAAGTAGCATGGCGGGTTAATCATTAACTACAAGGAACCCCTAGTGATGGAGTTGGCCACTCCCTCTCTGCGCGCTCGCTCGCTCACTGAGGCCGGGCGACCAAAGGTCGCCCGACGCCCGGGCTTTGCCCGGGCGGCCTCAGTGAGCGAGCGAGCGCGCAGCCTTAATTAACCTAATTCACTGGCCGTCGTTTTACAACGTCGTGACTGGGAAAACCCTGGCGTTACCCAACTTAATCGCCTTGCAGCACATCCCCCTTTC |
| **AAV-miR-133-inhibitor** | CTGCGCGCTCGCTCGCTCACTGAGGCCGCCCGGGCAAAGCCCGGGCGTCGGGCGACCTTTGGTCGCCCGGCCTCAGTGAGCGAGCGAGCGCGCAGAGAGGGAGTGGCCAACTCCATCACTAGGGGTTCCTTGTAGTTAATGATTAACCCGCCATGCTACTTATCTACCAGGGTAATGGGGATCCTCTAGAACTATAGCTAGAATTCGCCCTTACGGGCCCATACAATTGGAGGGCCTATTTCCCATGATTCCTTCATATTTGCATATACGATACAAGGCTGTTAGAGAGATAATTGGAATTAATTTGACTGTAAACACAAAGATATTAGTACAAAATACGTGACGTAGAAAGTAATAATTTCTTGGGTAGTTTGCAGTTTTAAAATTATGTTTTAAAATGGACTATCATATGCTTACCGTAACTTGAAAGTATTTCGATTTCTTGGCTTTATATATCTTGTGGAAAGGACGAAACACCGGATCCGACGGCGCTAGGATCATCAACCAGCTGGTTGAAATCTGGGGACCAAACAAGTATTCTGGTCACAGAATACAACCAGCTGGTTGAAATCTGGGGACCAAACAAGATGATCCTAGCGCCGTCTTTTTTGGGCCCCCCCTCGAGGTCGGGATAAAAGCAGTCTGGGCTTTCACATGACAGCATCTGGGGCTGCGGCAGAGGGTCGGGTCCGAAGCGCTGCCTTATCAGCGTCCCCAGCCCTGGGAGGTGACAGCTGGCTGGCTTGTGTCAGCCCCTCGGGCACTCACGTATCTCCGTCCGACGGGTTTAAAATAGCAAAACTCTGAGGCCACACAATAGCTTGGGCTTATATGGGCTCCTGTGGGGGAAGGGGGAGCACGGAGGGGGCCGGGGCCGCTGCTGCCAAAATAGCAGCTCACAAGTGTTGCATTCCTCTCTGGGCGCCGGGCACATTCCTGCTGGCTCTGCCCGCCCCGGGGTGGGCGCCGGGGGGACCTTAAAGCCTCTGCCCCCCAAGGAGCCCTTCCCAGACAGCCGCCGGCACCCACCGCTCCGTGGGACGATCCCCGAAGCTCTAGAGCTTTATTGCGGTAGTTTATCACAGTTAAATTGCTAACGCAGTCAGTGCTTCTGACACAACAGTCTCGAACTTAAGCTGCAGAAGTTGGTCGTGAGGCACTGGGCAGGTAAGTATCAAGGTTACAAGACAGGTTTAAGGAGACCAATAGAAACTGGGCTTGTCGAGACAGAGAAGACTCTTGCGTTTCTGATAGGCACCTATTGGTCTTACTGACATCCACTTTGCCTTTCTCTCCACAGGTGTCCACTCCCAGTTCAATTACAGCTCTTAAGGCTAGAGTACTTAATACGACTCACTATAGGCTAGCCTCGAGAATTCACGCGGGCCGCCATGGTGAGCAAGGGCGAGGAGCTGTTCACCGGGGTGGTGCCCATCCTGGTCGAGCTGGACGGCGACGTAAACGGCCACAAGTTCAGCGTGTCCGGCGAGGGCGAGGGCGATGCCACCTACGGCAAGCTGACCCTGAAGTTCATCTGCACCACCGGCAAGCTGCCCGTGCCCTGGCCCACCCTCGTGACCACCCTGACCTACGGCGTGCAGTGCTTCAGCCGCTACCCCGACCACATGAAGCAGCACGACTTCTTCAAGTCCGCCATGCCCGAAGGCTACGTCCAGGAGCGCACCATCTTCTTCAAGGACGACGGCAACTACAAGACCCGCGCCGAGGTGAAGTTCGAGGGCGACACCCTGGTGAACCGCATCGAGCTGAAGGGCATCGACTTCAAGGAGGACGGCAACATCCTGGGGCACAAGCTGGAGTACAACTACAACAGCCACAACGTCTATATCATGGCCGACAAGCAGAAGAACGGCATCAAGGTGAACTTCAAGATCCGCCACAACATCGAGGACGGCAGCGTGCAGCTCGCCGACCACTACCAGCAGAACACCCCCATCGGCGACGGCCCCGTGCTGCTGCCCGACAACCACTACCTGAGCACCCAGTCCGCCCTGAGCAAAGACCCCAACGAGAAGCGCGATCACATGGTCCTGCTGGAGTTCGTGACCGCCGCCGGGATCACTCTCGGCATGGACGAGCTGTACAAGTAATAAGCTCGCGTGGTACCTCTAGAGTCGACCCGGGCGGCCTCGAGGACGGGGTGAACTACGCCTGAGGATCCGATCTTTTTCCCTCTGCCAAAAATTATGGGGACATCATGAAGCCCCTTGAGCATCTGACTTCTGGCTAATAAAGGAAATTTATTTTCATTGCAATAGTGTGTTGGAATTTTTTGTGTCTCTCACTCGGAAGCAATTCGTTGATCTGAATTTCGACCACCCATAATACCCATTACCCTGGTAGATAAGTAGCATGGCGGGTTAATCATTAACTACAAGGAACCCCTAGTGATGGAGTTGGCCACTCCCTCTCTGCGCGCTCGCTCGCTCACTGAGGCCGGGCGACCAAAGGTCGCCCGACGCCCGGGCTTTGCCCGGGCGGCCTCAGTGAGCGAGCGAGCGCGCAGCCTTAATTAACCTAATTCACTGGCCGTCGTTTTACAACGTCGTGACTGGGAAAACCCTGGCGTTACCCAACTTAATCGCCTTGCAGCACATCCCCCTTTCGCCAGCTGGCGTAATAGCGAAGAGGCCCGCACCGATCGCCCTTCCCAACAGTTGCGCAGCCTGAATGGCGAATGGGACGCGCCCTGTAGCGGCGCATTAAGCGCGGCGGGTGTGGTGGTTACGCGCAGCGTGACCGCTACACTTGCCAGCGCCCTAGCGCCCGCTCCTTTCGCTTTCTTCCCTTCCTTTCTCGCCACGTTCGCCGGCTTTCCCCGTCAAGCTCTAAATCGGGGGCTCCCTTTAGGGTTCCGATTTAGTGCTTTACGGCACCTCGACCCCAAAAAACTTGATTAGGGTGATGGTTCACGTAGTGGGCCATCGCCCTGATAGACGGTTTTTCGCCCTTTGACGTTGGAGTCCACGTTCTTTAATAGTGGACTCTTGTTCCAAACTGGAACAACACTCAACCCTATCTCGGTCTATTCTTTTGATTTATAAGGGATTTTGCCGATTTCGGCCTATTGGTTAAAAAATGAGCTGATTTAACAAAAATTTAACGCGAATTTTAACAAAATATTAACGCTTACAATTTAGGTGGCACTTTTCGGGGAAATGTGCGCGGAACCCCTATTTGTTTATTTTTCTAAATACATTCAAATATGTATCCGCTCATGAGACAATAACCCTGATAAATGCTTCAATAATATTGAAAAAGGAAGAGTATGAGTATTCAACATTTCCGTGTCGCCCTTATTCCCTTTTTTGCGGCATTTTGCCTTCCTGTTTTTGCTCACCCAGAAACGCTGGTGAAAGTAAAAGATGCTGAAGATCAGTTGGGTGCACGAGTGGGTTACATCGAACTGGATCTCAACAGCGGTAAGATCCTTGAGAGTTTTCGCCCCGAAGAACGTTTTCCAATGATGAGCACTTTTAAAGTTCTGCTATGTGGCGCGGTATTATCCCGTATTGACGCCGGGCAAGAGCAACTCGGTCGCCGCATACACTATTCTCAGAATGACTTGGTTGAGTACTCACCAGTCACAGAAAAGCATCTTACGGATGGCATGACAGTAAGAGAATTATGCAGTGCTGCCATAACCATGAGTGATAACACTGCGGCCAACTTACTTCTGACAACGATCGGAGGACCGAAGGAGCTAACCGCTTTTTTGCACAACATGGGGGATCATGTAACTCGCCTTGATCGTTGGGAACCGGAGCTGAATGAAGCCATACCAAACGACGAGCGTGACACCACGATGCCTGTAGCAATGGCAACAACGTTGCGCAAACTATTAACTGGCGAACTACTTACTCTAGCTTCCCGGCAACAATTAATAGACTGGATGGAGGCGGATAAAGTTGCAGGACCACTTCTGCGCTCGGCCCTTCCGGCTGGCTGGTTTATTGCTGATAAATCTGGAGCCGGTGAGCGTGGGTCTCGCGGTATCATTGCAGCACTGGGGCCAGATGGTAAGCCCTCCCGTATCGTAGTTATCTACACGACGGGGAGTCAGGCAACTATGGATGAACGAAATAGACAGATCGCTGAGATAGGTGCCTCACTGATTAAGCATTGGTAACTGTCAGACCAAGTTTACTCATATATACTTTAGATTGATTTAAAACTTCATTTTTAATTTAAAAGGATCTAGGTGAAGATCCTTTTTGATAATCTCATGACCAAAATCCCTTAACGTGAGTTTTCGTTCCACTGAGCGTCAGACCCCGTAGAAAAGATCAAAGGATCTTCTTGAGATCCTTTTTTTCTGCGCGTAATCTGCTGCTTGCAAACAAAAAAACCACCGCTACCAGCGGTGGTTTGTTTGCCGGATCAAGAGCTACCAACTCTTTTTCCGAAGGTAACTGGCTTCAGCAGAGCGCAGATACCAAATACTGTTCTTCTAGTGTAGCCGTAGTTAGGCCACCACTTCAAGAACTCTGTAGCACCGCCTACATACCTCGCTCTGCTAATCCTGTTACCAGTGGCTGCTGCCAGTGGCGATAAGTCGTGTCTTACCGGGTTGGACTCAAGACGATAGTTACCGGATAAGGCGCAGCGGTCGGGCTGAACGGGGGGTTCGTGCACACAGCCCAGCTTGGAGCGAACGACCTACACCGAACTGAGATACCTACAGCGTGAGCTATGAGAAAGCGCCACGCTTCCCGAAGGGAGAAAGGCGGACAGGTATCCGGTAAGCGGCAGGGTCGGAACAGGAGAGCGCACGAGGGAGCTTCCAGGGGGAAACGCCTGGTATCTTTATAGTCCTGTCGGGTTTCGCCACCTCTGACTTGAGCGTCGATTTTTGTGATGCTCGTCAGGGGGGCGGAGCCTATGGAAAAACGCCAGCAACGCGGCCTTTTTACGGTTCCTGGCCTTTTGCTGGCCTTTTGCTCACATGTTCTTTCCTGCGTTATCCCCTGATTCTGTGGATAACCGTATTACCGCCTTTGAGTGAGCTGATACCGCTCGCCGCAGCCGAACGACCGAGCGCAGCGAGTCAGTGAGCGAGGAAGCGGAAGAGCGCCCAATACGCAAACCGCCTCTCCCCGCGCGTTGGCCGATTCATTAATGCAGCTGGCACGACAGGTTTCCCGACTGGAAAGCGGGCAGTGAGCGCAACGCAATTAATGTGAGTTAGCTCACTCATTAGGCACCCCAGGCTTTACACTTTATGCTTCCGGCTCGTATGTTGTGTGGAATTGTGAGCGGATAACAATTTCACACAGGAAACAGCTATGACCATGATTACGCCAGATTTAATTAAGGCCTTAATTAGGGCCAGCTGGCGTAATAGCGAAGAGGCCCGCACCGATCGCCCTTCCCAACAGTTGCGCAGCCTGAATGGCGAATGGGACGCGCCCTGTAGCGGCGCATTAAGCGCGGCGGGTGTGGTGGTTACGCGCAGCGTGACCGCTACACTTGCCAGCGCCCTAGCGCCCGCTCCTTTCGCTTTCTTCCCTTCCTTTCTCGCCACGTTCGCCGGCTTTCCCCGTCAAGCTCTAAATCGGGGGCTCCCTTTAGGGTTCCGATTTAGTGCTTTACGGCACCTCGACCCCAAAAAACTTGATTAGGGTGATGGTTCACGTAGTGGGCCATCGCCCTGATAGACGGTTTTTCGCCCTTTGACGTTGGAGTCCACGTTCTTTAATAGTGGACTCTTGTTCCAAACTGGAACAACACTCAACCCTATCTCGGTCTATTCTTTTGATTTATAAGGGATTTTGCCGATTTCGGCCTATTGGTTAAAAAATGAGCTGATTTAACAAAAATTTAACGCGAATTTTAACAAAATATTAACGCTTACAATTTAGGTGGCACTTTTCGGGGAAATGTGCGCGGAACCCCTATTTGTTTATTTTTCTAAATACATTCAAATATGTATCCGCTCATGAGACAATAACCCTGATAAATGCTTCAATAATATTGAAAAAGGAAGAGTATGAGTATTCAACATTTCCGTGTCGCCCTTATTCCCTTTTTTGCGGCATTTTGCCTTCCTGTTTTTGCTCACCCAGAAACGCTGGTGAAAGTAAAAGATGCTGAAGATCAGTTGGGTGCACGAG TGGGTTACATCGAACTGGATCTCAACAGCGGTAAGATCCTTGAGAGTTTTCGCCCCGAAGAACGTTTTCCAATGATGAGCACTTTTAAAGTTCTGCTATGTGGCGCGGTATTATCCCGTATTGACGCCGGGCAAGAGCAACTCGGTCGCCGCATACACTATTCTCAGAATGACTTGGTTGAGTACTCACCAGTCACAGAAAAGCATCTTACGGATGGCATGACAGTAAGAGAATTATGCAGTGCTGCCATAACCATGAGTGATAACACTGCGGCCAACTTACTTCTGACAACGATCGGAGGACCGAAGGAGCTAACCGCTTTTTTGCACAACATGGGGGATCATGTAACTCGCCTTGATCGTTGGGAACCGGAGCTGAATGAAGCCATACCAAACGACGAGCGTGACACCACGATGCCTGTAGCAATGGCAACAACGTTGCGCAAACTATTAACTGGCGAACTACTTACTCTAGCTTCCCGGCAACAATTAATAGACTGGATGGAGGCGGATAAAGTTGCAGGACCACTTCTGCGCTCGGCCCTTCCGGCTGGCTGGTTTATTGCTGATAAATCTGGAGCCGGTGAGCGTGGGTCTCGCGGTATCATTGCAGCACTGGGGCCAGATGGTAAGCCCTCCCGTATCGTAGTTATCTACACGACGGGGAGTCAGGCAACTATGGATGAACGAAATAGACAGATCGCTGAGATAGGTGCCTCACTGATTAAGCATTGGTAACTGTCAGACCAAGTTTACTCATATATACTTTAGATTGATTTAAAACTTCATTTTTAATTTAAAAGGATCTAGGTGAAGATCCTTTTTGATAATCTCATGACCAAAATCCCTTAACGTGAGTTTTCGTTCCACTGAGCGTCAGACCCCGTAGAAAAGATCAAAGGATCTTCTTGAGATCCTTTTTTTCTGCGCGTAATCTGCTGCTTGCAAACAAAAAAACCACCGCTACCAGCGGTGGTTTGTTTGCCGGATCAAGAGCTACCAACTCTTTTTCCGAAGGTAACTGGCTTCAGCAGAGCGCAGATACCAAATACTGTTCTTCTAGTGTAGCCGTAGTTAGGCCACCACTTCAAGAACTCTGTAGCACCGCCTACATACCTCGCTCTGCTAATCCTGTTACCAGTGGCTGCTGCCAGTGGCGATAAGTCGTGTCTTACCGGGTTGGACTCAAGACGATAGTTACCGGATAAGGCGCAGCGGTCGGGCTGAACGGGGGGTTCGTGCACACAGCCCAGCTTGGAGCGAACGACCTACACCGAACTGAGATACCTACAGCGTGAGCTATGAGAAAGCGCCACGCTTCCCGAAGGGAGAAAGGCGGACAGGTATCCGGTAAGCGGCAGGGTCGGAACAGGAGAGCGCACGAGGGAGCTTCCAGGGGGAAACGCCTGGTATCTTTATAGTCCTGTCGGGTTTCGCCACCTCTGACTTGAGCGTCGATTTTTGTGATGCTCGTCAGGGGGGCGGAGCCTATGGAAAAACGCCAGCAACGCGGCCTTTTTACGGTTCCTGGCCTTTTGCTGGCCTTTTGCTCACATGTTCTTTCCTGCGTTATCCCCTGATTCTGTGGATAACCGTATTACCGCCTTTGAGTGAGCTGATACCGCTCGCCGCAGCCGAACGACCGAGCGCAGCGAGTCAGTGAGCGAGGAAGCGGAAGAGCGCCCAATACGCAAACCGCCTCTCCCCGCGCGTTGGCCGATTCATTAATGCAGCTGGCACGACAGGTTTCCCGACTGGAAAGCGGGCAGTGAGCGCAACGCAATTAATGTGAGTTAGCTCACTCATTAGGCACCCCAGGCTTTACACTTTATGCTTCCGGCTCGTATGTTGTGTGGAATTGTGAGCGGATAACAATTTCACACAGGAAACAGCTATGACCATGATTACGCCAGATTTAATTAAGGCCTTAATTAGG |

**Table S2. PCR primers.**

| Nppa | Forward, 5′-TTCCTCGTCTTGGCCTTTTG-3;  Reverse, 5′-CCTCATCTTCTACCGGCATC-3 |
| --- | --- |
| Nppb | Forward, 5′- GTCCAGCAGAGACCTCAAAA-3;  Reverse, 5′- AGGCAGAGTCAGAAACTGGA-3 |
| Ctgf | Forward, 5′- GGGCCTCTTCTGCGATTTC-3;  Reverse, 5′- ATCCAGGCAAGTGCATTGGTA-3 |
| GapDH | Forward, 5′-AGGTCGGTGTGAACGGATTTG-3;  Reverse, 5′- TGTAGACCATGTAGTTGAGGTCA-3 |

Data S1. miR-133 predicted targets based on miRDB database.

Data S2. miR-133 predicted targets based on TargetScan database.

Data S3. The Python codes for modeling a cardiomyocyte as a cuboid (120×20×20 μm) and calculated whether the whole cardiomyocyte or at least the center of the CM should be within the radius of 84.5 μm to the margins of the infected.

Data S4. The Python codes for modeling a cardiomyocyte as a cuboid (120×20×20 μm) and calculated whether the whole cardiomyocyte or at least the half of a cell should be within the radius of 84.5 μm to the margins of the infected.
