# Supplementary material for: Non-cell-autonomous manner of AAV administration to attenuate cardiomyocyte hypertrophy by targeting paracrine signaling on ECM to reduce viral dosage
Source: Signal Transduct Target Ther. 2022 Jan 3;7:2. doi: 10.1038/s41392-021-00715-z (PMC8724271; doi:10.1038/s41392-021-00715-z)
Supplement: Supplementary file 6 — English editing certification [file 41392_2021_715_MOESM6_ESM.pdf]

Charlesworth Author Services

EDITORIAL CERTIFICATE

This document certifies that the manuscript below was edited for correct English language usage, grammar, punctuation and spelling by qualified native English speaking editors at Charlesworth Author Services.

Paper Title:

A modified AAV delivery strategy to attenuate cardiomyocyte hypertrophy with reduced viral dosage via targeting ECM

Author:

Yifei Li

Date certificate issued:

Tuesday 19th January, 2021

www.cwauthors.com
